# Timepoint-Specific Percentile Reference Curves for C-Reactive Protein and Erythrocyte Sedimentation Rate After Uncomplicated Primary Reverse Shoulder Arthroplasty: A Single-Center Cohort Study

**DOI:** 10.3390/diagnostics16182993

**Published:** 2026-09-16

**Authors:** Jaemin Lee, Seung Gyu Yang, Doo Sup Kim

**Affiliations:** Department of Orthopedic Surgery, Wonju College of Medicine, Yonsei University, Wonju 26426, Republic of Korea; megatal12@yonsei.ac.kr (J.L.); sgyang0202@yonsei.ac.kr (S.G.Y.)

**Keywords:** reverse shoulder arthroplasty, C-reactive protein, erythrocyte sedimentation rate, percentile reference curve, normative reference values, periprosthetic joint infection, postoperative trajectory

## Abstract

**Background/Objectives:** No shoulder-specific percentile reference range exists for postoperative C-reactive protein (CRP) or erythrocyte sedimentation rate (ESR) after reverse shoulder arthroplasty (RSA); clinicians rely on time-invariant thresholds borrowed from lower-extremity cohorts, and the ESR trajectory is uncharacterized. **Methods:** We retrospectively studied a single-surgeon registry of elective primary RSA (2018–2026); after predefined exclusions, 214 procedures (206 patients) remained, with no infection events by design. CRP and ESR were measured preoperatively and on postoperative days 2, 5, and 14 (exploratory days 1, 3, 7). We computed empirical 50th–95th percentile curves with bootstrap confidence intervals, supported by mixed-effects, quantile-regression, missingness, and within-patient fold-change analyses. **Results:** CRP peaked on day 2 (95th percentile 13.33 mg/dL); its day 14 95th percentile returned to, and lay numerically below, the preoperative 95th percentile (1.53 versus 2.34 mg/dL). ESR fell on day 2 (77.8% of procedures at or below their preoperative value), then rose and remained elevated at day 14 (95th percentile 56.00 mm/h); upper percentiles exceeded the 30 mm/h threshold at baseline and from day 3 onward. **Conclusions:** These curves describe an uncomplicated course rather than validated diagnostic thresholds; they provide a shoulder-specific normative reference and ESR trajectory for RSA, and a benchmark for future infection-event cohorts.

## 1. Introduction

Reverse shoulder arthroplasty (RSA) has expanded rapidly in shoulder reconstruction, with indications now spanning cuff tear arthropathy, complex proximal humeral fractures, and revision of failed anatomic implants. Periprosthetic joint infection (PJI), although uncommon—contemporary registry data place primary shoulder arthroplasty PJI incidence at approximately 1% to 3% [1]—remains among the most consequential complications, imposing a substantial burden through extended hospitalization, staged revision, and protracted antibiotic therapy [2,3,4]. Diagnosis is complicated by lower-virulence microbiology, in which the absence of overt systemic signs is the rule rather than the exception [1]. Serum C-reactive protein (CRP) and erythrocyte sedimentation rate (ESR) remain the first-line inflammatory markers worldwide, routinely used by 93% and 68% of shoulder surgeons, respectively [5].

The diagnostic performance of these markers is markedly attenuated in the shoulder relative to the hip and knee. A 2025 meta-analysis of 89 studies reported pooled sensitivities of 79% for CRP and 76% for ESR at conventional cutoffs but found shoulder-specific data insufficient for stratified analysis [6], and a shoulder-focused meta-analysis reported a pooled sensitivity of only 0.14 for elevated CRP or ESR in established shoulder PJI [7]. At the conventional 2018 International Consensus Meeting (ICM) cutoff of 10 mg/L, approximately 42% of ICM-defined infections remained below threshold [8], and a revision cohort reported preoperative CRP and ESR sensitivities of 67% and 65% [9]. Yet shoulder-specific reference curves remain sparse. Two cohorts—a prospective series of 58 patients [10] and a retrospective series of 280 [11]—described the postoperative CRP course, reproducing an early peak and approximate normalization, but reported mean trends rather than percentile reference curves; neither characterized the corresponding ESR trajectory, which, to our knowledge, has not previously been described after shoulder arthroplasty. The challenge is compounded by the predominance of *Cutibacterium acnes*, in which high deep-tissue loads occur without a sinus tract or wound drainage [12], conventional virulence markers do not distinguish ICM-definite from probable infection [13], and many culture-positive cases occur without an inflammatory host response [14]. A time-resolved, percentile-based reference range may therefore provide an alternative interpretive framework to a single time-invariant threshold, pending prospective validation.

We characterized the postoperative trajectory of serum CRP and ESR after uncomplicated primary RSA performed for elective degenerative indications—cuff tear arthropathy, massive irreparable rotator cuff tear, and primary glenohumeral osteoarthritis—and derived timepoint-specific percentile reference curves (50th, 75th, 90th, and 95th) at the preoperative window and postoperative days (POD) 1, 2, 3, 5, 7, and 14, with the preoperative baseline and the routine postoperative windows (POD 2, POD 5, POD 14) supporting the primary reference and the non-routine windows (POD 1, POD 3, POD 7) reported as exploratory. Non-elective indications were excluded a priori because their distinct baseline inflammatory state would distort a pooled reference. We additionally examined whether diagnosis group, implant manufacturer, age, and sex modified the trajectories, framing the curves as a single-center normative benchmark for future infection-event cohorts and external validation rather than a validated diagnostic threshold.

## 2. Materials and Methods

### 2.1. Study Design and Setting

We conducted a retrospective single-center single-surgeon cohort study at a tertiary referral hospital between 1 January 2018 and 22 April 2026, reported in accordance with the Strengthening the Reporting of Observational Studies in Epidemiology (STROBE) recommendations for cohort studies [15]. The Institutional Review Board of Wonju Severance Christian Hospital approved the protocol (IRB No. CR326065) and waived written informed consent given the retrospective design and exclusive use of de-identified electronic medical record (EMR) data.

### 2.2. Participants

Eligible patients were identified from a surgeon-maintained operative registry updated prospectively at each procedure. Inclusion criteria were a primary RSA performed for an elective degenerative indication (cuff tear arthropathy, massive irreparable rotator cuff tear without arthritis, or primary glenohumeral osteoarthritis); age 50 years or older; a preoperative CRP or ESR value within 90 days before or on the day of surgery; and CRP or ESR available at a minimum of one early postoperative timepoint among POD 1, 2, 3, 5, 7, and 14.

We restricted the cohort to elective degenerative indications for reasons of estimand definition rather than of clinical importance. Non-elective indications—acute proximal humerus fracture, post-traumatic sequelae, and arthroplasty after prior septic arthritis—represent a substantial share of contemporary RSA practice and are arguably the settings in which a postoperative inflammatory reference would be most useful, since early postoperative inflammation is hardest to interpret there. They were nonetheless deferred for three reasons. First, each shifts the preoperative acute-phase reactant distribution upward, so a pooled preoperative percentile would describe no single clinical state and the within-patient change from it would not be comparable across strata. Second, soft-tissue injury and operative exposure differ from elective degenerative surgery, so pooling would inflate the variance of the reference distribution and widen every percentile interval without indicating which stratum the widening came from. Third, a pooled curve cannot be disaggregated after the fact except by indication-stratified estimation, which requires per-stratum denominators at each timepoint that a consecutive single-surgeon series was not designed to supply. We therefore treat the non-elective population as a separate estimand, and a dedicated analysis of the trauma and post-traumatic cohort, with its own baseline and percentile curves, is planned as the next stage of this work.

Patients were excluded for any of the following: (1) revision RSA, analyzed separately; (2) non-elective or non-degenerative indication (acute proximal humerus fracture, post-traumatic sequelae, avascular necrosis, inflammatory arthritis, tumor or soft-tissue mass, or prior septic shoulder); (3) ambiguous operative coding not confirmed as RSA on chart review; (4) any infection event within 90 days, defined as surgical site infection by Centers for Disease Control and Prevention criteria or PJI by the 2018 ICM shoulder-specific definition [16]; (5) active autoimmune disease known to elevate baseline acute-phase reactants; (6) simultaneous non-shoulder surgical procedures; (7) concurrent active infection at the time of surgery; (8) active oncologic treatment within 12 months before surgery; (9) chronic kidney disease with an estimated glomerular filtration rate below 30 mL/min/1.73 m^2^; and (10) absence of both baseline CRP and ESR values. An alternative periprosthetic-infection definition, whose adequacy in the shoulder has recently been debated [17], was not adopted to maintain consistency with the contemporary shoulder-specific consensus. Criterion 2 was applied by hand-curation of the registry at enrollment and verified at analysis against operative records and implant sticker logs; the remaining criteria were applied at analysis by EMR query and chart re-review. A final analytic cohort of 214 primary RSA procedures in 206 patients was retained. Infection-free status was ascertained by chart review through 1 June 2026; the six most recent procedures (operated March–April 2026) had fewer than 90 days of follow-up at this census, so their 90-day infection-free status was not yet fully confirmed. Patients with bilateral staged RSA contributed each side as a separate surgical event, with within-patient correlation accommodated in the modeling stage. Three patients with mixed or atypical indications were retained at the operating surgeon’s discretion and grouped descriptively as “Other,” a stratum too small (*n* = 3) to be modeled separately. Degenerative cases carrying secondary trauma- or osteonecrosis-related annotations were retained when chart review confirmed an elective degenerative primary indication and were classified by that primary indication.

### 2.3. Definition of “Uncomplicated”

Throughout this report, “uncomplicated” denotes the absence of the prespecified infection and inflammatory exclusion criteria (criteria 4, 5, 7, and 9)—that is, a postoperative course free of surgical site infection and PJI—and not an entirely uneventful recovery. Events that can elicit a systemic acute-phase response without constituting surgical site infection or PJI were deliberately not made exclusion criteria: superficial wound drainage, delayed wound healing, postoperative hematoma, heterotopic ossification, and non-infectious fever were retained, as were non-surgical-site inflammatory events such as urinary tract infection, pneumonia, and venous thromboembolism. Retaining them was intentional, because the comparator relevant to a postoperative measurement is the population without periprosthetic infection—which necessarily includes minor and non-infectious complications—rather than an idealized subset with a perfectly uneventful course. Such events may therefore contribute to the upper percentiles, and the curves should be read as describing the post-RSA state without surgical site infection or PJI.

### 2.4. Surgical Procedure and Perioperative Protocol

One fellowship-trained shoulder surgeon performed every procedure through a standardized deltopectoral approach in the beach-chair position, with a mechanical limb positioner (Spider; TENET Medical Engineering, Calgary, AB, Canada) used in every case. A single perioperative care pathway governed the inpatient course, with planned discharge on POD 5. Intravenous tranexamic acid (TXA), which reduces perioperative blood loss in shoulder arthroplasty [18], was not administered routinely; an exception was granted only when chronic antiplatelet therapy could not be withheld. A closed-suction drain was placed in every case and removed on POD 2. Antibiotic prophylaxis and venous thromboembolism prevention were identical across the cohort.

### 2.5. Variables

Patient-level variables included age at surgery, sex, body mass index (BMI), smoking status, primary diagnosis, and comorbidities relevant to the inflammatory response. The single procedure with no recorded smoking-status entry was coded as never-smoker, consistent with the institutional admission-form workflow; the same procedure lacked structured entries for five comorbidity-history fields (hypertension, diabetes mellitus, cancer, tuberculosis, and hepatitis), which were coded as absent on the same basis. Anthropometric fields from the anesthesia record were incomplete in four procedures (height available in 211 and weight and BMI in 210 of 214), and the corresponding baseline summaries were computed on an available-case basis. Cuff tear arthropathy was distinguished from massive irreparable rotator cuff tear by the operating surgeon on the Hamada classification [19], recorded as a free-text indication label and mapped to the analysis groups by an automated keyword rule; independent re-reading was not performed, the diagnosis-stratified analysis being secondary and exploratory. Surgical variables comprised operative side, anesthesia type and duration, implant manufacturer and product, and humeral and glenoid fixation method. Implant manufacturer, product line, and cementless-versus-cemented fixation were extracted by structured review of intraoperative implant sticker photographs, with discordances reconciled by author review and one indeterminate humeral-fixation label coded as cementless per the institutional default; a de-identified per-procedure audit is provided as Supplementary Table S8.

The biomarkers of interest were serum CRP (mg/dL; values reported in mg/L were converted at extraction, 1.0 mg/dL = 10 mg/L) and ESR (mm/h), both assayed throughout the study period in the institution’s single hospital-based central laboratory—CRP by a latex-enhanced immunoturbidimetric assay on an automated analyzer (Cobas 8000, Roche Diagnostics, Mannheim, Germany; institutional upper reference value 0.5 mg/dL), and ESR by the automated Westergren method (institutional reference range 0–15 mm/h for men and 0–20 mm/h for women). The assay platform and reference ranges were unchanged across the study period, so no cross-laboratory harmonization was required. Measurements were retrieved preoperatively and at POD 1, 2, 3, 5, 7, and 14; the institutional protocol did not sample beyond POD 14, consistent with the prior shoulder arthroplasty CRP normalization study [10]. Each timepoint was operationalized with a prespecified window (POD 1, 12–36 h; POD 2, 36–60 h; POD 3, 60–84 h; POD 5, 4–6 days; POD 7, 7–9 days; POD 14, 12–17 days); when more than one value fell within a postoperative window, the measurement closest to the nominal day was used. For the preoperative window only, every eligible measurement was retained, so the preoperative denominator (269 for CRP, 250 for ESR) exceeds the cohort size of 214 procedures. Total white blood cell count and hemoglobin were extracted as candidate covariates but were not modeled, with the analysis confined to CRP and ESR.

### 2.6. Blood Sampling Protocol and Analytic Denominators

CRP and ESR were ordered together as a single inflammatory panel on POD 2, POD 5, and POD 14, as part of the standing postoperative order set applied to every patient on the single perioperative pathway. The panel was routine rather than triggered by clinical suspicion, and no clinical criterion—fever, wound appearance, or otherwise—had to be met for a patient to be sampled. POD 2 and POD 5 were inpatient draws, with planned discharge on POD 5; the observed postoperative day of discharge was 5.5 ± 2.7 days (median 5, IQR 5–5; range 3–30) across the 213 procedures with an interpretable discharge date. The POD 14 panel was, by contrast, an outpatient follow-up draw obtained at the first scheduled clinic visit, and it therefore depended on the patient returning to this institution. POD 1, POD 3, and POD 7 did not form part of the standing order set, were sampled at the treating team’s discretion, and are designated non-routine throughout. Hematologic indices followed a separate and, in the immediate postoperative period, denser schedule, a complete blood count being obtained on the day of surgery and on POD 1 in addition to the protocol days; the sparse POD 1 CRP and ESR denominators therefore reflected which assays were ordered that day rather than the absence of a venipuncture, whereas at POD 3 and POD 7 most procedures had no blood sampling within the window at all.

Because the analyzed per-timepoint denominators at the routine windows (171 to 183 for CRP and 153 to 181 for ESR) are smaller than the cohort of 214 procedures, we audited every window against the full cohort and report the accounting as Supplementary Table S9. Each unavailable observation was assigned to one of three mutually exclusive categories, in priority order: the protocol assay was recovered in an adjacent window, meaning that the draw drifted and the measurement remains in the reference curve one window over (a category defined for the routine windows only, the non-routine windows having no protocolized draw that could drift); blood was drawn within the window, but the panel did not include that assay; or no blood sampling occurred within the window at all, in which case procedures at the routine windows additionally had no assay in either neighboring window, adjacent recovery being assessed first. The audit is reported in the Results and motivated the POD 14 missingness sensitivity analysis described below.

### 2.7. Outcomes

The primary outcome was the timepoint-specific percentile reference curves of CRP and ESR (50th, 75th, 90th, and 95th percentiles) across the preoperative baseline and POD 1, 2, 3, 5, 7, and 14. Secondary outcomes were the effects of diagnosis group, implant manufacturer, age dichotomized at 70 years (a conventional threshold for older adult arthroplasty recipients, although the cohort’s older age distribution left few patients below it), and sex on the trajectories. TXA use (one of 214 procedures) and humeral stem fixation (205 cementless versus nine cemented, confounded with vendor) were treated outside the formal subgroup framework: the former precluded any contrast, and the latter was evaluated by a post hoc Mann–Whitney comparison at the preoperative window and the three routine postoperative timepoints (POD 2, POD 5, and POD 14). Because exclusion criterion 4 removed all 90-day infection events, the descriptive infection-comparison framework was retained as a template for prospective application to future external infection-event cohorts.

### 2.8. Sample Size

No a priori sample-size calculation was performed, and no conventional power calculation was applicable to the primary estimand. The cohort is the complete consecutive single-surgeon experience over more than eight years rather than a sample drawn to a target: every eligible procedure in the study period was included, so no recruitment decision existed that a power calculation could have informed, and no minimum required sample was predefined. The primary estimand is also a descriptive percentile rather than a difference tested against a null hypothesis, so the conventional inputs to a power calculation—an anticipated effect size and an accepted type II error rate—have no counterpart in this design.

Adequacy is instead governed by the precision of the percentile estimate, which we report directly: every percentile estimated from five or more observations carries a non-parametric bootstrap 95% confidence interval (CI; Supplementary Table S7). At the routine windows the intervals are narrow (POD 2 CRP 95th percentile 13.33 mg/dL, 95% CI 10.65–14.58); at the non-routine windows the same machinery makes the opposite point (POD 3 CRP 95th percentile 9.65 mg/dL, 95% CI 7.31–20.08), and the single window with fewer than five observations (POD 1 ESR, *n* = 2) is reported without an interval. The analysis is likewise conditioned on the sample obtained: the percentiles are empirical and non-parametric and impose no distributional form, the denominator accompanies every estimate, and a prespecified *n* < 30 threshold flags estimates as exploratory; no predefined minimum sample per window was set. For scale, each routine window rests on more than 150 measurements for both markers (CRP 171, 174, and 183; ESR 153, 174, and 181), whereas every non-routine window rests on fewer than 40.

### 2.9. Statistical Analysis

Baseline characteristics were summarized as mean ± standard deviation (SD) or median [interquartile range, IQR] for continuous variables, based on distributional inspection and the Anderson–Darling test for samples with *n* ≥ 50 (Shapiro–Wilk otherwise), and as *n* (%) for categorical variables. Because CRP is typically right-skewed, log-transformed values [log(CRP + 0.1)] were used for parametric modeling, whereas raw percentiles were used for the reference curves.

Three complementary approaches were prespecified. Approach A computed the 50th, 75th, 90th, and 95th percentiles empirically at each timepoint, with non-parametric percentile bootstrap 95% CIs (B = 2000 replicates, seed = 42) wherever *n* ≥ 5; the single window with *n* < 5 (POD 1 ESR, *n* = 2) was reported without an interval, and intervals at timepoints with *n* < 20 are subject to tail instability. Routine windows (POD 2, POD 5, POD 14) were distinguished from non-routine windows (POD 1, POD 3, POD 7) throughout. Approach B fitted a linear mixed-effects model (LMM) with the biomarker (log-transformed CRP, untransformed ESR) as the outcome, timepoint as a categorical fixed effect referenced to baseline, and a per-patient random intercept; a random slope was not specified because the sparsely sampled non-routine windows rendered it non-identifiable. Estimated marginal means were back-transformed for CRP and reported with 95% CIs. ESR was modeled on its native scale for direct clinical interpretability; because Approach B is corroborative, the Gaussian approximation for the right-skewed ESR distribution was considered acceptable. Approach C fitted quantile regression at the 95th percentile with timepoint as a categorical predictor, as a sensitivity benchmark for the empirical 95th-percentile curve.

Subgroup analyses added time × covariate interaction terms to the LMM, each assessed by a likelihood-ratio test (LRT) against a model omitting the interaction. The mixed-effects models were estimated by restricted maximum likelihood; for the interaction likelihood-ratio tests, the full and reduced models were refitted by maximum likelihood, as required for valid comparison of nested models that differ in fixed effects. The significance level was *p* < 0.05 (two-sided), with Bonferroni correction across the eight prespecified time × subgroup interaction tests (four factors × two biomarkers; α = 0.05/8 = 0.00625). Tests in which the full interaction model failed to reach a non-singular optimum under any optimizer attempted (a singular Hessian) yielded no valid chi-square deviate and are reported as non-estimable rather than as LRT = 0.00 with *p* = 1.00; the corresponding subgroups were characterized descriptively. Per-test optimizer, convergence, Hessian status, log-likelihoods, and degrees of freedom for all eight tests are reported in Supplementary Table S6. The post hoc humeral fixation comparison comprised eight independent two-sample Mann–Whitney tests (four timepoints × two biomarkers) reported with uncorrected *p*-values; at the preoperative window, which retained all eligible baseline draws, this comparison was observation-level rather than procedure-level. The CRP LMM was fitted with the Powell optimizer after the default L-BFGS-B returned a singular Hessian; the ESR model converged on the default.

### 2.10. Within-Patient Change from the Individual Preoperative Baseline

Because preoperative values vary between patients, we additionally characterized the magnitude and the rate of change from each patient’s own baseline. This analysis was added post hoc at the revision stage in response to peer review; it was not prespecified, and it is reported as descriptive rather than confirmatory.

For each procedure, the baseline was that patient’s own last preoperative draw of the corresponding marker. At every postoperative window with a paired value, we computed the fold change (postoperative divided by preoperative value), the absolute change (postoperative minus preoperative value, in mg/dL for CRP and mm/h for ESR), and the velocity (absolute change divided by the nominal postoperative day). Only procedures contributing both a preoperative and a timepoint value entered a given window. Fold change was summarized as the median [IQR] with the 90th and 95th percentiles, absolute change as the median [IQR], and velocity as the median; we additionally report the proportion of procedures at or below that patient’s own preoperative value. Because the preoperative CRP distribution lies close to the assay reporting floor, a ratio taken against it has a near-zero denominator and is numerically unstable; the absolute change is therefore treated as the primary within-patient summary for CRP, whereas preoperative ESR lies well above its measurement floor and its ratio is interpreted alongside the absolute change (Supplementary Table S11).

### 2.11. Missing Data

Approach A used an available-case strategy with the denominator reported for each estimate. The LMM handled unbalanced repeated measures under a missing-at-random assumption without imputation; because the non-routine windows were sampled at the treating team’s discretion, missingness there may be informative, and those estimates are reported as descriptive. Multiple imputation was not performed.

The POD 14 window warranted separate examination because—unlike the drift-dominated POD 2 shortfall—its unavailable observations largely represent an outpatient draw that never occurred, a mechanism that could plausibly depend on the patient’s postoperative course. We therefore compared procedures that contributed a POD 14 CRP measurement (*n* = 183) with those that did not (*n* = 31) across 17 baseline, perioperative, and early postoperative variables, chosen to include those most directly informative about the unobserved value (the POD 2 and POD 5 CRP concentrations, its closest available proxies), the postoperative day of discharge, which is the mechanism by which an outpatient draw would most plausibly be missed, and the characteristics that define the reference population. Each variable was assessed by the standardized mean difference (SMD), with |SMD| < 0.10 taken as negligible imbalance, alongside a two-sided test selected by variable type: Mann–Whitney U for continuous variables, chi-square for categorical variables, and Fisher’s exact test where any observed cell count was below five. No multiplicity correction was applied, as the analysis was intended to be sensitive to imbalance rather than protected against false positives. It was performed post hoc at the revision stage and is reported as a sensitivity analysis rather than a confirmatory test of an a priori hypothesis (Supplementary Table S10).

### 2.12. Software

Analyses were performed in Python 3.11.9 with pandas 3.0.2, statsmodels 0.14.6 (mixed-effects models and quantile regression), scipy 1.17.1, numpy 2.4.4, and matplotlib 3.10.8 with seaborn 0.13.2. The analyses added at revision (Supplementary Tables S10–S12) were performed in the same environment, and all resampling used seed = 42.

## 3. Results

### 3.1. Patient Flow and Baseline Characteristics

The surgeon-curated registry of 218 elective primary RSA procedures was re-audited at analysis: three procedures were reclassified as anatomic total shoulder arthroplasty and one as a revision RSA and were excluded (Supplementary Figure S1). No procedure met exclusion criteria 4 to 10; chart review of all 218 procedures identified no surgical site infection or PJI event meeting criterion 4. The final analytic cohort comprised 214 uncomplicated primary RSA procedures in 206 patients, of whom eight underwent staged bilateral RSA. Baseline demographic, clinical, and surgical characteristics—including the implant-manufacturer distribution and the predominantly cementless humeral and uniformly cementless glenoid fixation—are presented in Table 1.

### 3.2. Sampling Density and Denominators

A per-window audit of the analytic denominator against the full cohort of 214 procedures is presented in Supplementary Table S9 and shows two mechanisms that differ in kind. At POD 2 the shortfall was predominantly sampling-day drift rather than patient attrition: of the 43 procedures without a CRP value inside the window, 34 had the protocol assay recovered in the adjacent POD 1 or POD 3 window and therefore remained within the reference curve one window over, and only nine had no early CRP assay at all; of the 61 without a POD 2 ESR value, 32 were recovered in an adjacent window, and the remaining 29 had no early ESR value—19 after an in-window venipuncture whose panel omitted ESR and 10 with no blood sampling in the window. Pooled across the early windows (POD 1–3), CRP was available in 205 of 214 procedures (95.8%) and ESR in 185 of 214 (86.4%), and at least one postoperative CRP value was available in every procedure and at least one postoperative ESR value in 212 of 214 (99.1%). The POD 14 shortfall was of a different character, that draw being an outpatient sample obtained after a planned POD 5 discharge: 183 procedures (85.5%) contributed a POD 14 CRP value and 181 (84.6%) an ESR value, and of the 31 without a POD 14 CRP value, 27 had no assay in the POD 14 window or in the adjacent POD 7 window and none had a CRP measurement anywhere between POD 10 and POD 30, indicating the absence of an outpatient draw rather than a scheduled draw that drifted. The sparse POD 1 denominators reflected assay ordering rather than absence of venipuncture, a complete blood count having been drawn in nearly every procedure on that day (white blood cell count and hemoglobin available in 212 of 214, 99.1%) without CRP or ESR on the panel. At POD 3 and POD 7, the mechanism differed, the great majority of procedures having had no blood sampling within the window at all (169 of 180 and 199 of 202 CRP-missing procedures, respectively); nearly all of these nonetheless contributed CRP values at the neighboring routine windows, so they are sparsely sampled on these days rather than absent from the reference curve.

### 3.3. CRP Trajectory

**Figure 1:**
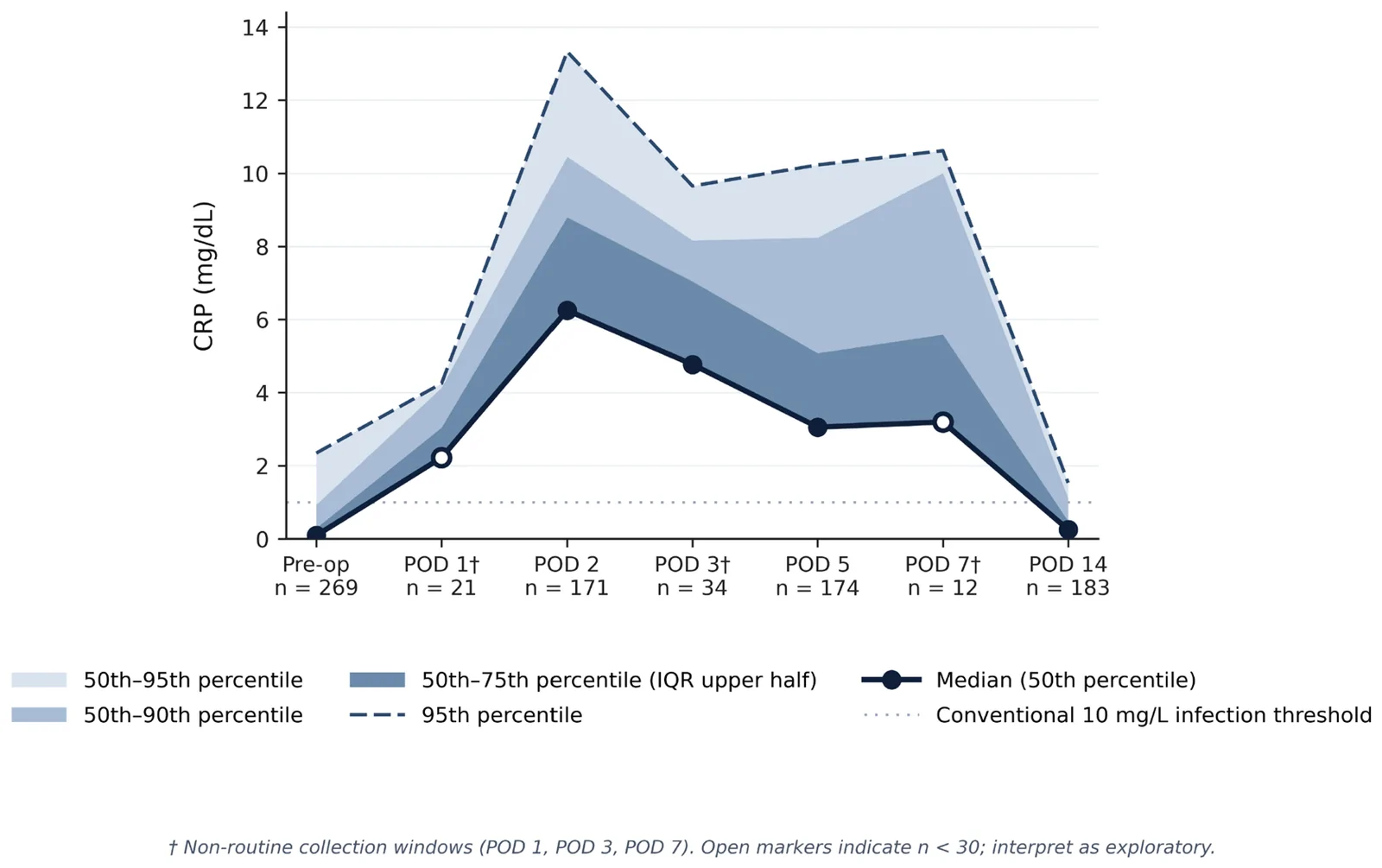
CRP trajectory after uncomplicated primary reverse shoulder arthroplasty (*N* = 214 procedures in 206 patients). Per-timepoint serum C-reactive protein (CRP, mg/dL) across the preoperative window and postoperative days 1 through 14. The solid line is the median; shaded envelopes show the 50th–75th, 50th–90th, and 50th–95th percentile ranges; the dashed line traces the 95th percentile; and the dotted horizontal line marks the conventional 10 mg/L (1.0 mg/dL) periprosthetic infection threshold. Sample sizes are annotated beneath each timepoint; daggered timepoints (POD 1, POD 3, POD 7) are non-routine windows, and open median markers (POD 1, POD 7) fall below the prespecified *n* < 30 caution threshold and are exploratory. CRP, C-reactive protein; POD, postoperative day.

Per-timepoint sample sizes and percentile values for CRP are presented in Table 2 and Figure 1. Median CRP rose from the preoperative baseline to a peak at POD 2 and declined across the subsequent routine windows, remaining above the preoperative median at POD 14. By POD 14 the 95th percentile of CRP had returned to, and lay numerically below, the preoperative 95th percentile (1.53 versus 2.34 mg/dL); the preoperative 95th percentile fell within the POD 14 bootstrap 95% CI, and the two intervals (preoperative 1.63–3.24 mg/dL; POD 14 1.18–2.53 mg/dL) overlapped across 1.63–2.53 mg/dL (Supplementary Table S7). The upper tail of the CRP distribution had thus resolved to its preoperative range by the end of the second postoperative week, whereas the center of the distribution had not, with the POD 14 median remaining above the preoperative median (0.25 versus 0.09 mg/dL). The mixed-effects model recovered the central CRP trajectory, and the 95th-percentile quantile regression was concordant with the empirical 95th-percentile curve (Supplementary Table S1).

**Table 2:** CRP percentile reference curves (uncomplicated RSA).

Timepoint	*n*	Median (IQR)	75th	90th	95th	Mean ± SD	Range
Pre-op	269	0.09 (0.05–0.29)	0.29	0.93	2.34	0.43 ± 0.97	0.01–8.03
POD 1 †	21	2.22 (0.70–3.04)	3.04	4.13	4.25	2.12 ± 1.47	0.02–4.78
POD 2	171	6.25 (4.33–8.79)	8.79	10.45	13.33	6.68 ± 3.27	0.03–20.90
POD 3 †	34	4.77 (2.40–7.04)	7.04	8.17	9.65	5.11 ± 3.68	0.98–20.08
POD 5	174	3.06 (1.66–5.08)	5.08	8.24	10.23	3.94 ± 3.24	0.03–19.77
POD 7 †	12	3.19 (1.24–5.59)	5.59	10.00	10.62	4.15 ± 3.59	0.10–10.98
POD 14	183	0.25 (0.12–0.48)	0.48	1.11	1.53	0.54 ± 1.12	0.02–12.15

Values in mg/dL. *n* = procedures contributing one measurement to the given postoperative timepoint (closest-to-nominal-day draw selected as described in the Methods); at the preoperative window (−90 to 0 days), all eligible draws are retained, so a procedure with multiple preoperative measurements contributes more than one observation and n exceeds the cohort size. † Non-routine collection windows (not part of the institutional protocol; sampled on a case-by-case basis). Per-window *n* is materially smaller than at the routine POD 2, POD 5, and POD 14 windows, and POD 1 and POD 7 in particular fall below the prespecified n < 30 caution threshold defined in the Methods. Percentile estimates at these timepoints are reported for completeness and should be interpreted as exploratory rather than as definitive reference values. Non-parametric bootstrap 95% confidence intervals for the 50th, 75th, 90th, and 95th percentiles at every timepoint are provided in Supplementary Table S7. Abbreviations: CRP, C-reactive protein; POD, postoperative day; IQR, interquartile range; SD, standard deviation.

### 3.4. ESR Trajectory

**Figure 2:**
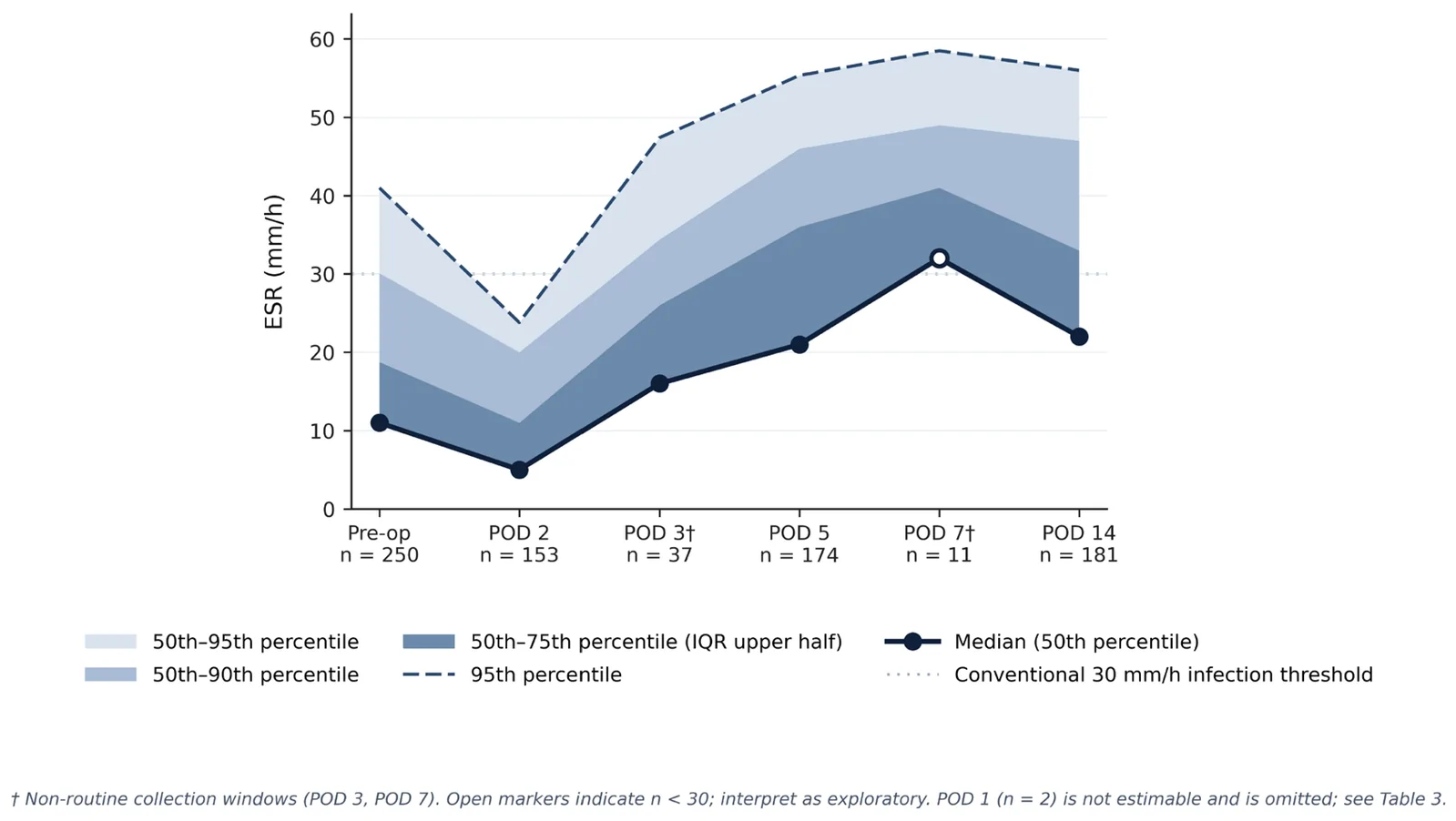
ESR trajectory after uncomplicated primary reverse shoulder arthroplasty (*N* = 214 procedures in 206 patients). Per-timepoint erythrocyte sedimentation rate (ESR, mm/h) across the preoperative window and postoperative days 2 through 14. The solid line is the median; shaded envelopes show the 50th–75th, 50th–90th, and 50th–95th percentile ranges; the dashed line traces the 95th percentile; and the dotted horizontal line marks the conventional 30 mm/h periprosthetic infection threshold. Sample sizes are annotated beneath each timepoint; daggered timepoints (POD 3, POD 7) are non-routine windows, and the open median marker (POD 7) falls below the prespecified *n* < 30 caution threshold and is exploratory. The POD 1 window contributed only two ESR measurements, from which a median, an interquartile range, and upper percentiles cannot be derived; that window is therefore reported as not estimable in Table 3 and is omitted from this figure rather than drawn from an interpolation. ESR, erythrocyte sedimentation rate; POD, postoperative day.

Per-timepoint sample sizes and percentile values for ESR are presented in Table 3 and Figure 2. Median ESR was lower than the preoperative median at POD 2, rose through POD 5 and POD 7, and remained above the preoperative median at POD 14. The upper percentile curves exceeded the conventional 30 mm/h periprosthetic infection threshold at the preoperative window and from POD 3 through POD 14 but lay below it at POD 2; percentiles were not estimable at POD 1, where only two procedures contributed an ESR value. The mixed-effects model recovered the same central ESR trajectory—a POD 2 nadir, a rise through POD 5 and POD 7, and persistent elevation at POD 14—and the 95th-percentile quantile regression was concordant with the empirical 95th-percentile curve at the routine timepoints, diverging upward at the sparsely sampled non-routine windows (Supplementary Table S1); as central-tendency estimates, the marginal means remained below the 30 mm/h comparator at the routine timepoints.

**Table 3:** ESR percentile reference curves (uncomplicated RSA).

Timepoint	*n*	Median (IQR)	75th	90th	95th	Mean ± SD	Range
Pre-op	250	11.00 (5.00–18.75)	18.75	30.10	41.00	15.20 ± 15.24	2.00–91.00
POD 1 †‡	2	NE	NE	NE	NE	NE	2.00–23.00
POD 2	153	5.00 (2.00–11.00)	11.00	20.00	23.80	7.76 ± 7.75	2.00–42.00
POD 3 †	37	16.00 (11.00–26.00)	26.00	34.40	47.40	19.70 ± 14.40	2.00–63.00
POD 5	174	21.00 (10.00–36.00)	36.00	46.00	55.35	24.13 ± 16.70	2.00–71.00
POD 7 †	11	32.00 (16.00–41.00)	41.00	49.00	58.50	30.09 ± 20.07	2.00–68.00
POD 14	181	22.00 (11.00–33.00)	33.00	47.00	56.00	23.63 ± 16.32	2.00–76.00

Values in mm/h. *n* = procedures contributing one measurement to the given postoperative timepoint (closest-to-nominal-day draw selected as described in the Methods); at the preoperative window (−90 to 0 days) all eligible draws are retained, so a procedure with multiple preoperative measurements contributes more than one observation and n exceeds the cohort size. † Non-routine collection windows (not part of the institutional protocol; sampled on a case-by-case basis). Per-window n is materially smaller than at the routine POD 2, POD 5, and POD 14 windows, and POD 1 and POD 7 in particular fall below the prespecified *n* < 30 caution threshold defined in the Methods. Percentile estimates at these timepoints are reported for completeness and should be interpreted as exploratory rather than as definitive reference values. ‡ The POD 1 ESR window contains only two observations (2.00 and 23.00 mm/h). Percentiles and a dispersion estimate cannot be meaningfully derived from two values, so they are reported as not estimable (NE) rather than printed; the denominator and the two observed values are given here so that the sampling record remains complete. This window was likewise non-estimable for bootstrap intervals. Bootstrap 95% confidence intervals for all other timepoints and percentiles are provided in Supplementary Table S7. Abbreviations: NE, not estimable; ESR, erythrocyte sedimentation rate; POD, postoperative day; IQR, interquartile range; SD, standard deviation.

### 3.5. Within-Patient Change from Individual Baseline

Change from each patient’s own last preoperative measurement, expressed as a fold change, an absolute change, and a velocity per postoperative day, is presented in Supplementary Table S11. A preoperative CRP value was available for all 214 procedures (median 0.08 mg/dL) and a preoperative ESR value for 213 (median 10.0 mm/h). The ratio metric behaved differently for the two markers, and the difference is attributable to the baseline distribution rather than to the postoperative response. Because the median preoperative CRP lay essentially at the assay reporting floor, the CRP fold change carried a near-zero denominator and was correspondingly dispersed (POD 2 median 63.83, IQR 24.30–127.31, 95th percentile 303), whereas the absolute rise at the same window was tightly grouped (median 5.97 mg/dL, IQR 3.96–8.18); for CRP, the absolute value is therefore the more stable reference quantity. For ESR, whose preoperative distribution sits well above the assay floor, the ratio was well behaved—1.78 [0.91–3.74] at POD 5 and 2.00 [1.15–3.29] at POD 14.

Two further observations emerged at the individual level. First, 77.8% of the 153 procedures with paired preoperative and POD 2 ESR values had a POD 2 ESR at or below the patient’s own preoperative value, confirming within patients the paradoxical POD 2 decline that the group percentile curve could describe but not establish. Second, by POD 14, 25.1% of procedures had returned to or below the patient’s own preoperative CRP against 21.0% for ESR despite the earlier POD 2 ESR nadir, reinforcing the divergent recovery kinetics of the two markers.

### 3.6. Missingness Sensitivity Analysis

Procedures that contributed a POD 14 CRP value (*n* = 183) were compared with those that did not (*n* = 31) across 17 variables spanning demographics, comorbidity, diagnosis, implant and fixation characteristics, anesthesia duration, postoperative day of discharge, and preoperative and early postoperative biomarker values, with SMDs reported alongside each test (Supplementary Table S10). Only the postoperative day of discharge differed significantly: procedures that did not contribute a POD 14 value had a longer stay (6.8 ± 4.7 versus 5.3 ± 2.1 days; *p* < 0.001; the discharge date was interpretable in 213 of the 214 procedures). The direction of that difference is opposite to the informative-missingness mechanism of principal concern, in which patients retained in hospital for clinical reasons would be preferentially sampled; here the unsampled procedures stayed longer, a pattern consistent with transfer or convalescence elsewhere and therefore with no outpatient draw at our institution. POD 2 CRP (*p* = 0.271) and POD 5 CRP (*p* = 0.242), the closest available proxies for the unobserved POD 14 value, did not differ significantly, although several variables carried standardized imbalance at or above the 0.10 threshold (POD 2 CRP |SMD| = 0.200; age 0.202; cuff tear arthropathy diagnosis 0.258), so a modest degree of informative missingness cannot be excluded on distributional grounds. The largest imbalance was in smoking status (|SMD| = 0.438), driven entirely by none of the 31 unsampled procedures being a current or former smoker, compared with 16 of 183 (8.7%) among the sampled; the difference was not significant (*p* = 0.136) and reflects a sparsely populated stratum.

### 3.7. Subgroup Analyses

**Figure 3:**
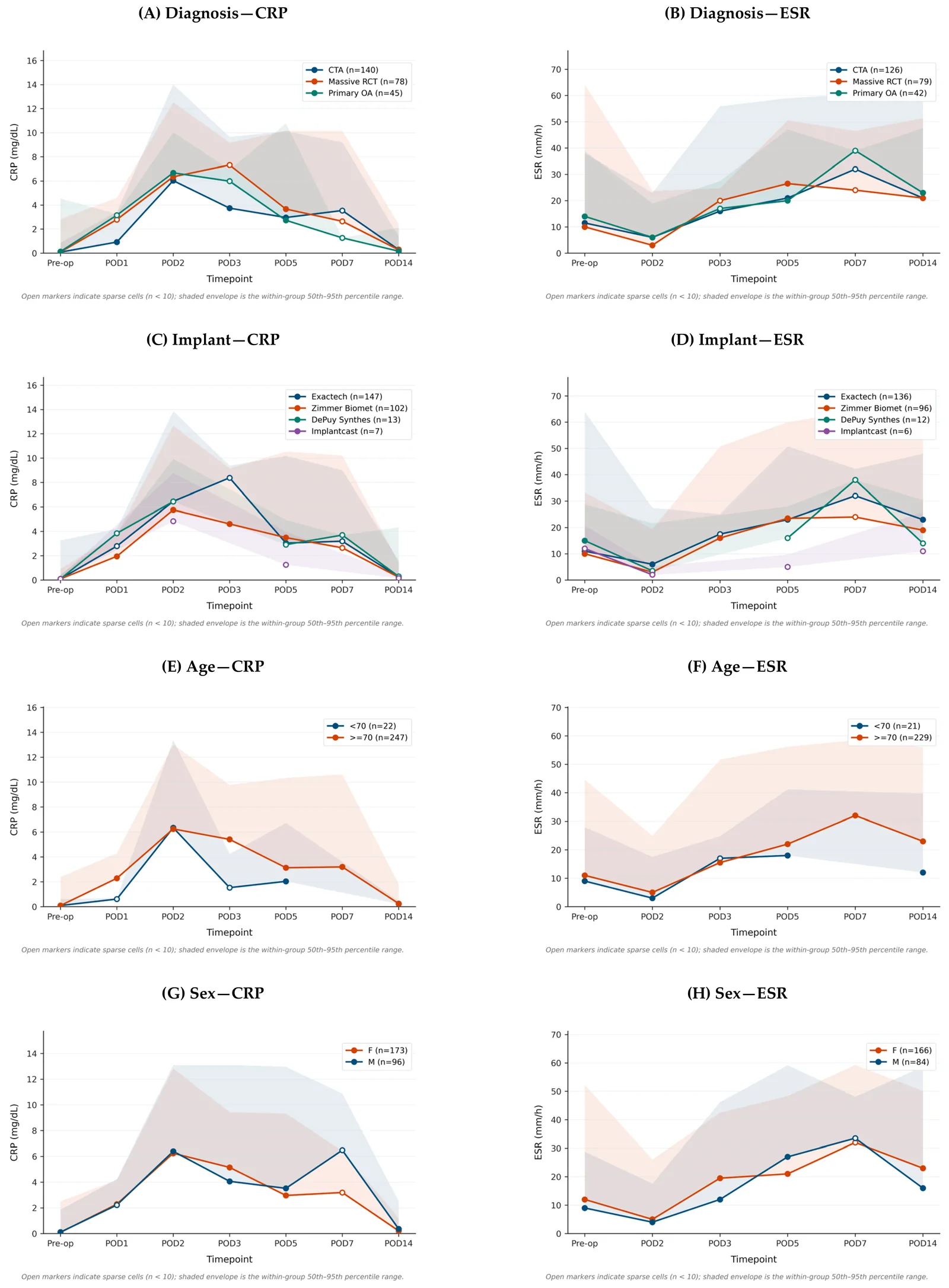
Subgroup-stratified biomarker trajectories. Within-stratum median curves, each with its 50th–95th percentile range shown as a shaded envelope, stratified by diagnosis group (panels (**A**,**B**)), implant manufacturer (panels (**C**,**D**)), age dichotomized at 70 years (panels (**E**,**F**)), and sex (panels (**G**,**H**)), with CRP in the left panels and ESR in the right. The three procedures grouped descriptively as “Other” (Table 1) are not shown as a diagnosis stratum, so panels (**A**,**B**) rest on slightly fewer procedures than Table 2 and Table 3. As in Figure 2 and Table 3, the POD 1 window—which contains only two ESR observations cohort-wide—is omitted from the ESR panels (**B**,**D**,**F**,**H**). In each panel legend, n is the largest number of measurements the stratum contributed at any single timepoint; because the preoperative window retains every eligible draw, these counts are observation-level and exceed the per-stratum procedure counts of Table 1. Open median markers denote within-stratum timepoints with *n* < 10 (a stratum-level convention distinct from the cohort-level *n* < 30 caution threshold defined in the Methods) and are exploratory. Six of the eight prespecified time × subgroup interaction tests were non-estimable (singular Hessian; Supplementary Table S6), and the two estimable tests (diagnosis group × CRP, sex × CRP) were non-significant. CRP, C-reactive protein; ESR, erythrocyte sedimentation rate.

Subgroup-stratified trajectories and time × subgroup interaction tests are presented in Figure 3 and Supplementary Tables S3–S7. Of the eight prespecified interaction tests, six were non-estimable owing to a singular Hessian under every optimizer attempted, reflecting small or sparsely populated strata for the implant-manufacturer and age interactions and an ESR-specific near-empty cell at POD 1 for the diagnosis-group and sex interactions; the two estimable tests, diagnosis group × CRP and sex × CRP, were non-significant against both the Bonferroni-adjusted and an uncorrected threshold (Supplementary Table S6). A post hoc, exploratory comparison of the cementless and cemented humeral fixation subsets did not detect a difference at the preoperative window or any of the three routine postoperative timepoints for either biomarker (all eight uncorrected Mann–Whitney tests non-significant: CRP *p* = 0.891, 0.893, 0.402, and 0.827 and ESR *p* = 0.157, 0.790, 0.175, and 0.494 at the preoperative, POD 2, POD 5, and POD 14 windows, respectively); with only nine cemented procedures this analysis is underpowered to exclude a clinically meaningful difference. Intravenous TXA was administered in one procedure and general anesthesia in every procedure, precluding an analytic contrast for either.

## 4. Discussion

### 4.1. Main Findings

In this single-surgeon cohort of 214 uncomplicated primary RSA procedures performed for elective degenerative indications, CRP peaked on POD 2 and had returned to its own preoperative distribution by POD 14: the POD 14 95th percentile (1.53 mg/dL) was numerically lower than the preoperative 95th percentile (2.34 mg/dL), which itself lay within the POD 14 bootstrap interval. The upper tail of the CRP distribution has therefore normalized by the end of the second postoperative week, whereas the center has not quite. This is the ordinary biology of postoperative CRP recovery and is described here as such; it is not evidence that these curves identify or exclude infection. ESR followed a kinetically distinct course, declining paradoxically on POD 2, ascending through POD 5 to POD 7, and remaining substantially elevated at POD 14, with its upper percentile curves exceeding the conventional 30 mm/h periprosthetic infection threshold at baseline and from POD 3 onward; a within-patient analysis added in revision (Supplementary Table S11) reproduced this divergence at the level of the individual procedure. Of the eight prespecified time × subgroup interaction tests, six were non-estimable and two non-significant, so the subgroup analyses are underpowered exploratory observations that neither confirm nor refute trajectory invariance.

### 4.2. Comparison with the Literature

To our knowledge, this provides the first percentile reference curves—rather than mean trends—for CRP, the first described postoperative ESR trajectory, and the largest paired CRP–ESR series after shoulder arthroplasty. Two prior cohorts described the postoperative CRP course without percentile curves: the prospective study of Torrens and colleagues followed 58 patients in a mixed-arthroplasty cohort and reported a POD 2 CRP peak with approximate normalization by POD 14 [10], and a retrospective series of 280 primary shoulder arthroplasties reported a POD 2 to 3 peak declining through POD 7, with reverse and stemmed implants peaking higher than stemless implants or hemiarthroplasty [11]. Both the POD 2 peak and the approximate POD 14 normalization were reproduced here, and the absence of a distinguishable cemented-versus-cementless difference accords with the reported lack of a bone-cement effect [11]. The present work additionally characterizes ESR, which prior shoulder arthroplasty cohorts did not report despite its routine use [5]. The CRP kinetics are concordant with the hip and knee literature, in which CRP peaks early and resolves within weeks [20,21,22,23], although the median peak was lower than the reported hip and knee peaks of approximately 116 to 140 mg/L [20]; ESR rose later and remained elevated through POD 14, in line with knee arthroplasty studies in which it stayed above baseline for several weeks [23]. The attenuated diagnostic performance of single-point CRP and ESR in shoulder PJI [6,8,24] reflects the predominance of low-virulence *Cutibacterium acnes*, in which conventional inflammatory markers and virulence features do not reliably separate infected from uninfected or ICM-definite from probable cases [12,13,25].

### 4.3. Clinical Implications

The percentile reference curves have two potential applications, given that the cohort contained no infection events and the curves describe uncomplicated postoperative values rather than validated diagnostic thresholds. As a contextual aid, the time-resolved 95th percentile provides a reference value against which a postoperative measurement can be compared at the timepoint it was drawn, in place of a time-invariant threshold. The preoperative 90th-percentile ESR curve already reached the conventional 30 mm/h threshold, so approximately one in ten preoperative measurements from these older, predominantly female candidates lay above the cutoff, limiting the usefulness of any single preoperative threshold. By POD 14 the upper tail of the CRP distribution had returned to its preoperative range, so a value above the reference band on that day is atypical for an uncomplicated course and may warrant attention, whereas a value inside the band is not reassurance, for the reasons set out in Section 4.4; a single ESR in the second postoperative week, by contrast, overlaps both the reference curve and the infection threshold and cannot, on its own, support a binary decision. As a research tool, the curves provide a shoulder-specific reference distribution against which infection-event trajectories can be benchmarked; because the dominant pathogen is indolent [1,12], whether such a kinetic reference adds diagnostic signal beyond a single threshold crossing remains to be tested in future infection-event cohorts. The reference is strengthened by a single surgeon and a single perioperative pathway—identical antibiotic prophylaxis, an institutional policy of withholding TXA, a recognized modifier of perioperative blood loss [18,26], and uniform drain and thromboembolism management—which removes a source of between-surgeon variability, and by the concordance of the mixed-effects model and quantile regression with the empirical percentiles at the protocolized timepoints.

Normalizing each postoperative measurement to the patient’s own preoperative value has been proposed as a potentially more informative metric than an absolute concentration, and the within-patient analysis performed in revision supports that proposal for ESR while qualifying it for CRP. Preoperative CRP sits at the assay reporting floor, so the ratio is formed over a near-zero denominator and becomes numerically unstable, whereas the corresponding absolute rise is tightly grouped; that dispersion is a property of the denominator rather than of the inflammatory response, and the absolute value therefore remains the more stable CRP reference. Preoperative ESR sits well above its floor, and the ratio behaves accordingly, so normalization to the patient’s own baseline is informative for ESR and is a reasonable metric to carry into prospective work. The same analysis supplies individual-level confirmation of the paradoxical POD 2 ESR decline, which the group-level curves could not establish.

### 4.4. Clinical Application and Caution

Reference curves derived from an infection-free cohort describe what happens in the absence of infection; by construction they say nothing about what infection looks like. These curves must therefore not be used to exclude periprosthetic joint infection, and least of all in the early postoperative period, when the operative acute-phase response is at its height and overlaps most heavily with any infective signal. A worked example makes the point concrete: a CRP of 8 mg/dL on POD 5 lies below the POD 5 95th percentile of 10.23 mg/dL and just below the 90th percentile of 8.24 mg/dL, so it falls inside the reference band and would be read as compatible with an uncomplicated course, yet the same value is entirely compatible with early infection. The reason is structural rather than a matter of estimation precision. Exclusion criterion 4 removed every 90-day infection event, so the cohort contains no infection events by design; in the absence of a comparator group with confirmed infection, the curves carry no information about how an infected trajectory would depart from them. No sensitivity, specificity, or likelihood ratio can be derived from these data, and none is reported.

It follows that a value falling inside the reference band must never be read as reassurance. The band is the range of values observed in patients who were not infected; it is not the range of values that excludes infection, and the two are not interchangeable. Because low-virulence organisms, *C. acnes* above all, frequently produce deep infection without a systemic inflammatory response [12,14], an unremarkable marker in a patient whose clinical picture is suggestive is uninformative rather than negative. Clinical context and physical examination—pain out of proportion, wound drainage or delayed healing, unexplained stiffness, early radiographic loosening—with aspiration and culture where indicated, take precedence over any single laboratory value; and where serial measurements exist, the shape of the trajectory is more informative than any one of them, so that a CRP failing to fall after its POD 2 peak, or falling and then rising again, warrants evaluation irrespective of whether each value lies inside the band. The appropriate use of these curves is therefore asymmetric: an atypical value for the day on which it was drawn may prompt closer scrutiny without in itself implying a diagnosis, whereas a typical value carries no complementary implication whatever.

### 4.5. Limitations

First, postoperative sampling density was non-uniform. POD 2, POD 5, and POD 14 correspond to the institutional protocol and are densely sampled, whereas POD 1, POD 3, and POD 7 were not and are sparse: CRP was available in 21, 34, and 12 procedures and ESR in 2, 37, and 11, respectively. The POD 1 ESR estimate, resting on two procedures, is essentially non-informative, and its percentiles are accordingly reported as not estimable. The timepoint audit added in revision shows that at POD 1 this sparsity reflects assay ordering rather than the absence of a blood draw, whereas at POD 3 and POD 7 most procedures had no blood sampling within the window at all; in either case ordering at the treating team’s discretion is itself a potentially informative mechanism, and the non-routine windows may over-represent procedures in which something prompted an additional test. These are precisely the days on which a clinician confronted with an unexpected fever or a wound concern would most want guidance, and the days on which this cohort has least to offer. Accordingly, no clinical decision should rest on the POD 1, POD 3, or POD 7 percentiles until they have been reproduced in substantially larger cohorts with protocolized sampling on those days.

Second, exclusion criterion 4 removed all 90-day infection events, so no internal comparator exists, and no receiver-operating-characteristic analysis could be performed. We regard the absence of even a small external or prospective proof-of-concept validation cohort as a genuine limitation of the present work rather than a matter to be deferred: these are reference values, not validated thresholds, and how they behave in patients who go on to develop infection is unknown. The six most recent procedures had fewer than 90 days of infection ascertainment at the data census, but contribute too small a fraction of the measurements at each routine timepoint to affect the percentile estimates materially. Third, “uncomplicated” here denotes the absence of surgical site infection and PJI and not an uneventful postoperative course, as defined in Section 2.3; the minor and non-infectious events retained under that definition can elicit a systemic acute-phase response and may therefore inflate the upper percentiles, most plausibly in the POD 5 and POD 14 windows.

Fourth, the cohort is narrow in ways that bound generalizability directly. It is single-surgeon, single-center, and single-country (Republic of Korea); the patients are older adults (mean age 78.4 ± 6.3 years, with only 18 procedures, 8.4%, in patients younger than 70 years) and predominantly female (141 of 214, 65.9%); and implant use is concentrated in two manufacturers (Exactech 54.2%, Zimmer Biomet 39.3%). These curves are consequently not universally applicable and should not be transported to other settings without external validation—specifically not to younger patients, not to other ethnic groups, and not to different implant designs, stemless RSA included, for which a lower postoperative CRP peak than that of stemmed implants has already been reported [11]. Because acute-phase reactant baselines rise with age, the reference values may in addition be shifted upward relative to younger cohorts; and although smoking is an independent risk factor for postoperative shoulder infection [27], the small smoker subgroup precluded a stable stratified estimate. The bootstrap confidence intervals quantify sampling variability within this cohort only and say nothing about the between-population differences at issue here.

Fifth, the absolute ESR is a composite measurement influenced by hemoglobin concentration, age, sex, and other physiological determinants of erythrocyte aggregation, so an ESR value carries less specific information about the acute-phase response than a CRP concentration of comparable rank. Hemoglobin was extracted as a candidate covariate but deliberately not modeled, the analysis having been confined to CRP and ESR; we therefore cannot adjust these curves for hemoglobin, nor quantify how far the sustained POD 5 to POD 14 ESR elevation reflects hemoglobin and demographic determinants rather than the acute-phase response itself. Referencing each value to the same patient’s own preoperative measurement partly mitigates this by holding the time-invariant determinants constant, but does not remove the effect of the postoperative fall in hemoglobin. Sixth, the reference does not extend beyond POD 14, so the ESR normalization tail is under-characterized. Seventh, the Hamada-based diagnosis label was assigned by the operating surgeon without independent radiographic re-reading, so the diagnosis subgroup analysis is exploratory.

Eighth, the subgroup analyses are underpowered and should be read as exploratory only. Six of the eight prespecified time × covariate interaction tests were non-estimable under a singular Hessian, and the two estimable tests were non-significant; a non-estimable test is an absence of information rather than evidence of trajectory invariance, so these data do not establish whether the curves apply equally across diagnosis groups, age strata, sexes, or implant manufacturers, and the stratified trajectories are reported descriptively with no inferential claim attached. The post hoc humeral fixation comparison is weaker still: with nine cemented procedures drawn predominantly from a single vendor, fixation and manufacturer cannot be separated, and the uniformly non-significant comparisons exclude nothing of clinical size. No a priori sample-size calculation was performed, and the achieved precision of each percentile estimate is therefore reported as its bootstrap confidence interval rather than asserted as attained power. TXA use, administered in a single procedure, and humeral stem fixation could not be entered into the mixed-effects framework.

### 4.6. Future Directions

The decisive next step is validation, stated in specific terms: a prospective, preferably multi-center series of primary RSA in which consecutive patients are sampled at the same prespecified windows used here, and infection events are adjudicated by the 2018 ICM shoulder-specific definition [16], so that infected and uninfected trajectories can be compared within one sampling frame, with the present curves entering as the prespecified reference. External application to an independent cohort containing infection events, including retrospective cohorts already holding serial markers, would provide a faster, albeit weaker, first test, and we regard even a small proof-of-concept cohort of this kind as a priority precisely because the present work lacks one. Until such a comparison exists, these curves remain descriptive reference values for the uncomplicated course and should not be adopted as a clinical surveillance tool. Three extensions follow directly from the analyses reported here: normalization of the ESR curves for hemoglobin, age, and sex; prospective testing of the within-patient ratio metric, which the present data indicate is informative for ESR but unstable for CRP; and extension to non-elective indications, to horizons beyond POD 14, and to the effects of stem fixation and TXA.

## 5. Conclusions

In uncomplicated primary reverse shoulder arthroplasty performed for elective degenerative indications, the timepoint-specific percentile reference curves derived from this 214-procedure single-surgeon cohort describe the C-reactive protein and erythrocyte sedimentation rate values compatible with an uncomplicated postoperative course: CRP had returned to, and lay numerically below, its preoperative 95th percentile by postoperative day 14 (1.53 versus 2.34 mg/dL), whereas ESR remained substantially elevated. Referencing each measurement to the patient’s own preoperative value reproduced this divergence within patients and showed that the postoperative day 2 ESR lay at or below that baseline in most procedures. A post hoc comparison showed no distinguishable CRP or ESR distribution between cementless and cemented humeral fixation, though the nine-procedure cemented stratum, predominantly from a single vendor, leaves this underpowered; the prespecified interaction tests were similarly exploratory (six non-estimable, two non-significant). No infection events occurred within the analytic cohort by construction, so these curves constitute a single-center normative reference and, to our knowledge, the first described ESR trajectory for RSA. They are a benchmark for future infection-event cohorts rather than validated diagnostic thresholds, and prospective validation will be required before adoption as a clinical surveillance tool.

## Figures and Tables

**Table 1:** Baseline characteristics (*N* = 214).

Variable	Value (*N* = 214)
Demographics	
Age (years)	78.4 ± 6.3
Age, median [IQR] (years)	78 [74–83]
Age, range (years)	61–93
Age < 70 years	18 (8.4%)
Sex—Female	141 (65.9%)
Sex—Male	73 (34.1%)
BMI (kg/m^2^; *n* = 210)	24.8 [22.6–27.1]
Height (cm; *n* = 211)	153.0 [149.0–160.3]
Weight (kg; *n* = 210)	59.5 [52.1–66.4]
Surgery	
Side—Right	143 (66.8%)
Side—Left	71 (33.2%)
Diagnosis	
CTA	115 (53.7%)
Massive RCT	62 (29.0%)
Primary OA	34 (15.9%)
Other	3 (1.4%)
ASA	
ASA 1	4 (1.9%)
ASA 2	98 (45.8%)
ASA 3	112 (52.3%)
ASA 4	0 (0.0%)
Comorbidity	
HTN	140 (65.4%)
DM	59 (27.6%)
CKD	9 (4.2%)
CVD	54 (25.2%)
Cancer (history)	4 (1.9%)
TB (history)	8 (3.7%)
Hepatitis (history)	4 (1.9%)
Lifestyle	
Smoking—current/ex	16 (7.5%)
Smoking—never	198 (92.5%)
Anesthesia	
Type—GA	214 (100.0%)
Duration (min)	130.0 [120.0–140.0]
Intraop	
TXA given	1 (0.5%)
Intraop transfusion	2 (0.9%)
Intake fluid (cc)	700.0 [600.0–900.0]
Implant	
Equinoxe Reverse Shoulder (Exactech, Gainesville, FL, USA)	116 (54.2%)
Comprehensive / Trabecular Metal Reverse (Zimmer Biomet, Warsaw, IN, USA)	84 (39.3%)
Delta Xtend (DePuy Synthes, Warsaw, IN, USA)	9 (4.2%)
Agilon (Implantcast, Buxtehude, Germany)	5 (2.3%)
Humeral stem fixation—cementless	205 (95.8%)
Humeral stem fixation—cemented	9 (4.2%)
Glenoid baseplate fixation—cementless	214 (100.0%)
Implant design variants	
Augmented glenoid baseplate (within cementless)	5 (2.3%)
Constrained humeral polyethylene liner	1 (0.5%)
Baseline labs	
Pre-op CRP (mg/dL)	0.1 [0.1–0.2]
Pre-op ESR (mm/h; *n* = 213)	10.0 [5.0–17.0]

Continuous variables: mean ± SD (normal) or median [IQR] (non-normal; Anderson–Darling/Shapiro–Wilk, α = 0.05). Categorical variables: *n* (%). Age is summarized as mean ± SD, the primary descriptor selected by the distributional test, with the median [IQR], the full range, and the number of procedures in patients younger than 70 years reported additionally to characterize the age distribution of the cohort. Default-coded counts (as detailed in the Methods): the “Smoking—never” count includes 1 procedure with no admission-form smoking entry folded into “never” per the institutional EMR default (observed-never = 197 + default-coded = 1 = 198); the same procedure lacked structured entries for the hypertension, diabetes, cancer, tuberculosis, and hepatitis history fields, which were likewise coded as absent; and the “Humeral stem fixation—cementless” count includes 1 procedure (No 104, Exactech Equinoxe) with an indeterminate humeral sticker label folded into “cementless” per the institutional default (observed-cementless = 204 + default-coded = 1 = 205). Anthropometric fields from the anesthesia record were incomplete: height was available for 211 and weight and BMI for 210 of the 214 procedures, and these rows are summarized over the available procedures. All other Table 1 variables have complete data (*N* = 214), except baseline ESR, which was available for 213 procedures (one procedure had a preoperative CRP but no preoperative ESR within the window). Baseline CRP and ESR (Table 1) summarize one preoperative value per procedure and therefore differ slightly from the preoperative percentile windows of Table 2 and Table 3, which retain every eligible preoperative draw (*n* = 269 for CRP and 250 for ESR). Abbreviations: SD, standard deviation; IQR, interquartile range; BMI, body mass index; CTA, cuff tear arthropathy; RCT, rotator cuff tear; OA, osteoarthritis; ASA, American Society of Anesthesiologists; HTN, hypertension; DM, diabetes mellitus; CKD, chronic kidney disease; CVD, cardiovascular disease; TB, tuberculosis; GA, general anesthesia; TXA, tranexamic acid; CRP, C-reactive protein; ESR, erythrocyte sedimentation rate.

## Data Availability

The data presented in this study are available on request from the corresponding author due to privacy and ethical restrictions.

## Supplementary Materials

The following supporting information can be downloaded at: https://www.mdpi.com/article/10.3390/diagnostics16182993/s1, Figure S1: STROBE participant flow diagram; Table S1: linear mixed-effects estimated marginal means and 95th-percentile quantile-regression estimates; Tables S2–S5: subgroup-stratified percentile trajectories; Table S6: linear mixed-effects convergence diagnostics; Table S7: non-parametric bootstrap 95% confidence intervals for the empirical percentile reference values; Table S8: de-identified per-procedure implant extraction audit; Table S9: timepoint denominator audit; Table S10: sensitivity analysis for informative missingness at postoperative day 14; Table S11: within-patient fold change and velocity relative to the individual preoperative baseline; Table S12: STROBE checklist.
