# Supplementary material for: Timepoint-Specific Percentile Reference Curves for C-Reactive Protein and Erythrocyte Sedimentation Rate After Uncomplicated Primary Reverse Shoulder Arthroplasty: A Single-Center Cohort Study
Source: Diagnostics (Basel). 2026 Sep 16;16(18):2993. doi: 10.3390/diagnostics16182993 (PMC13605674; doi:10.3390/diagnostics16182993)
Supplement: Supplementary file 1 [file diagnostics-16-02993-s001.zip › supplementary_materials_REV1_260822.pdf]

Supplementary Materials

Timepoint-Specific Percentile Reference Curves for C-Reactive Protein and Erythrocyte Sedimentation Rate After Uncomplicated Primary Reverse Shoulder Arthroplasty: A Single-Center Cohort Study

Revision 1 (REV1). Supplementary Tables S9-S11 are new at this revision and were added in response to the reviewers.

Supplementary Figure S1. STROBE Participant Flow

Patient inclusion and exclusion at successive stages from the master operative registry (N = 218 procedures initially listed as elective primary RSA) to the analytic cohort (N = 214 procedures in 206 patients). The master registry was hand-curated at the point of enrollment by chart review to exclude non-elective indications (criterion 2). Subsequent exclusions comprised implant-sticker reclassification (Nos 51, 71, 89 → anatomic total shoulder arthroplasty) and operative-record recoding (No 159 → revision RSA). RSA, reverse shoulder arthroplasty; CRP, C-reactive protein; ESR, erythrocyte sedimentation rate; SSI, surgical site infection; PJI, periprosthetic joint infection; eGFR, estimated glomerular filtration rate; STROBE, Strengthening the Reporting of Observational Studies in Epidemiology.

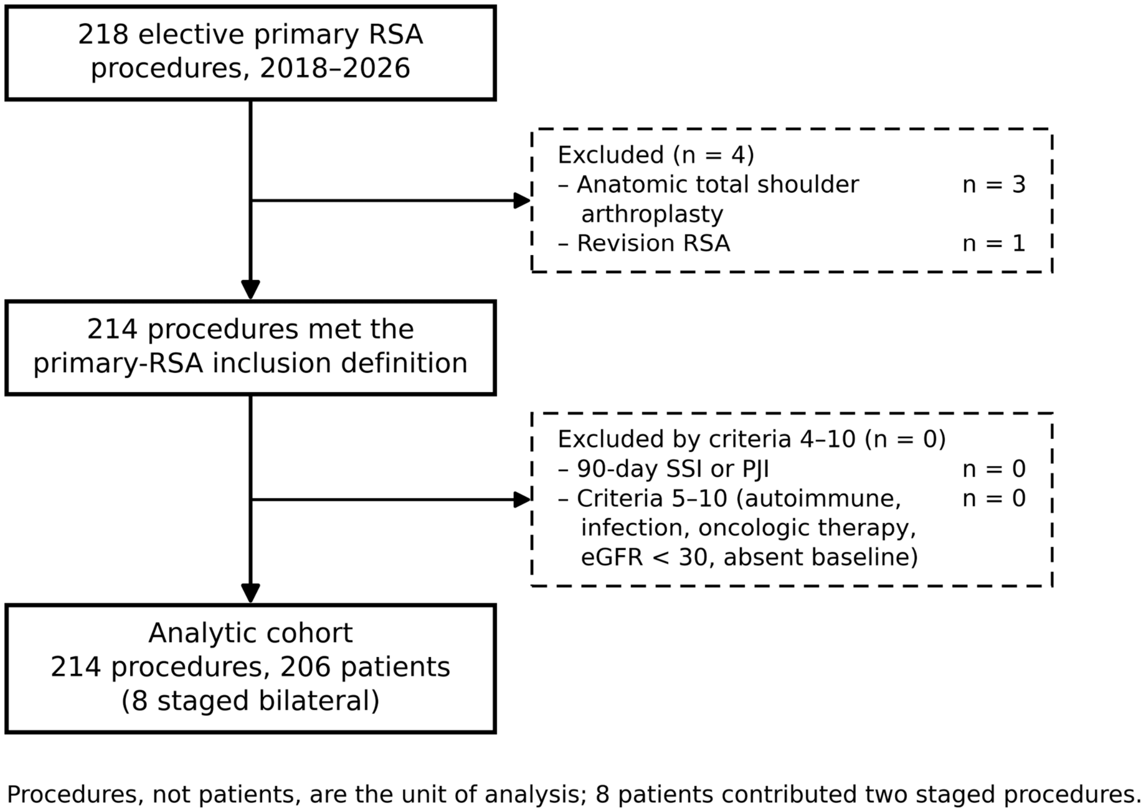

Supplementary Table S1. LMM EMM and Quantile Regression Estimates

Linear mixed-effects model (LMM) back-transformed estimated marginal means (EMM) with patient random intercept, and quantile-regression estimates at the 95th percentile, by timepoint. CRP estimates have been back-transformed from the log scale; ESR estimates are on the raw scale. Units: mg/dL for CRP, mm/h for ESR.

CRP — LMM EMM

Timepoint	EMM (mg/dL)	95% CI low	95% CI high	n
Pre-op	0.16	0.14	0.19	269
POD1	1.65	1.18	2.29	21
POD2	5.74	5.07	6.49	171
POD3	4.50	3.48	5.82	34
POD5	2.78	2.46	3.15	174
POD7	2.21	1.43	3.38	12
POD14	0.29	0.25	0.34	183

ESR — LMM EMM

Timepoint	EMM (mm/h)	95% CI low	95% CI high	n
Pre-op	13.94	11.99	15.89	250
POD1	5.10	-10.51	20.71	2
POD2	8.61	6.38	10.83	153
POD3	18.81	14.88	22.75	37
POD5	23.66	21.53	25.80	174
POD7	30.42	23.62	37.22	11
POD14	23.48	21.38	25.58	181

Quantile regression at the 95th percentile

Marker	Timepoint	q95	n
CRP	Pre-op	2.36	269
CRP	POD1	4.25	21
CRP	POD2	13.67	171
CRP	POD3	9.95	34
CRP	POD5	10.26	174
CRP	POD7	10.98	12
CRP	POD14	1.54	183
ESR	Pre-op	41.00	250
ESR	POD1	23.00	2
ESR	POD2	25.00	153
ESR	POD3	61.00	37
ESR	POD5	56.00	174
ESR	POD7	68.00	11
ESR	POD14	56.00	181

Estimates at the POD 1 ESR window (n = 2) are degenerate and shown for completeness only; consistent with the empirical-percentile handling (Methods; Table 3) they are regarded as non-estimable. The corresponding Gaussian linear mixed-effects 95% confidence interval extends below zero, a sparse-data artifact for a non-negative measure, and should be disregarded.

Supplementary Table S2. Diagnosis-Stratified Percentile Trajectories

Per-timepoint medians, 75th, 90th, and 95th percentiles (with per-stratum n) for CRP (mg/dL) and ESR (mm/h). Strata with fewer than five observations at a timepoint are degenerate and are shown for completeness only, not as estimates; this includes the POD 1 ESR window (n = 2), reported as not estimable in Table 3. The three procedures grouped descriptively as "Other" (Table 1) are not shown as a separate stratum, so the per-stratum denominators sum to slightly less than the corresponding totals in Tables 2 and 3.

CRP by Diagnosis group

Timepoint	Group	n	median	p75	p90	p95
Pre-op	CTA	140	0.08	0.22	0.49	0.86
Pre-op	Massive RCT	78	0.09	0.34	1.81	2.83
Pre-op	Primary OA	45	0.13	0.45	2.31	4.55
POD1	CTA	11	0.92	2.42	3.02	3.44
POD1	Massive RCT	7	2.79	4.19	4.46	4.62
POD1	Primary OA	2	3.15	3.21	3.25	3.26
POD2	CTA	87	6.03	8.91	10.61	14.01
POD2	Massive RCT	51	6.35	8.67	11.08	12.53
POD2	Primary OA	31	6.68	7.88	9.06	10.05
POD3	CTA	25	3.74	6.09	8.06	9.66
POD3	Massive RCT	4	7.32	7.91	8.86	9.17
POD3	Primary OA	4	5.99	6.30	6.79	6.95
POD5	CTA	92	2.96	4.78	8.29	10.13
POD5	Massive RCT	52	3.67	5.77	7.86	10.16
POD5	Primary OA	27	2.73	4.58	8.40	10.78
POD7	CTA	8	3.54	5.59	8.09	9.21
POD7	Massive RCT	3	2.65	6.82	9.31	10.15
POD7	Primary OA	1	1.27	1.27	1.27	1.27
POD14	CTA	95	0.29	0.48	0.90	1.38
POD14	Massive RCT	55	0.27	0.53	1.41	2.41
POD14	Primary OA	30	0.16	0.45	1.20	2.12

ESR by Diagnosis group

Timepoint	Group	n	median	p75	p90	p95
Pre-op	CTA	126	11.50	17.00	26.00	38.00
Pre-op	Massive RCT	79	10.00	20.00	33.00	64.20
Pre-op	Primary OA	42	14.00	22.75	36.40	38.90
POD1	CTA	1	2.00	2.00	2.00	2.00
POD1	Massive RCT	1	23.00	23.00	23.00	23.00
POD1	Primary OA	0				
POD2	CTA	81	6.00	13.00	21.00	23.00
POD2	Massive RCT	46	3.00	6.75	16.50	23.75

POD2	Primary OA	24	6.00	11.25	16.80	18.85
POD3	CTA	27	16.00	29.00	38.60	55.90
POD3	Massive RCT	5	20.00	20.00	23.60	24.80
POD3	Primary OA	4	17.00	21.50	26.00	27.50
POD5	CTA	94	21.00	35.00	46.00	59.00
POD5	Massive RCT	50	26.50	36.00	43.40	50.55
POD5	Primary OA	27	20.00	40.00	45.00	47.10
POD7	CTA	7	32.00	40.50	53.00	60.50
POD7	Massive RCT	3	24.00	36.50	44.00	46.50
POD7	Primary OA	1	39.00	39.00	39.00	39.00
POD14	CTA	95	21.00	33.00	48.60	61.00
POD14	Massive RCT	54	21.00	29.50	41.90	51.40
POD14	Primary OA	29	23.00	37.00	47.00	47.60

Supplementary Table S3. Implant-Manufacturer-Stratified Percentile Trajectories

Per-timepoint medians, 75th, 90th, and 95th percentiles (with per-stratum n) for CRP (mg/dL) and ESR (mm/h). Strata with fewer than five observations at a timepoint are degenerate and are shown for completeness only, not as estimates; this includes the POD 1 ESR window (n = 2), reported as not estimable in Table 3.

CRP by Implant company

Timepoint	Group	n	median	p75	p90	p95
Pre-op	Exactech	147	0.09	0.40	1.94	3.27
Pre-op	Zimmer Biomet	102	0.09	0.18	0.54	0.93
Pre-op	DePuy Synthes	13	0.09	0.16	0.44	0.46
Pre-op	Implantcast	7	0.10	0.24	0.32	0.36
POD1	Exactech	5	2.79	4.13	4.20	4.23
POD1	Zimmer Biomet	15	1.95	2.65	3.18	3.72
POD1	DePuy Synthes	1	3.85	3.85	3.85	3.85
POD1	Implantcast	0				
POD2	Exactech	108	6.46	8.88	10.16	13.87
POD2	Zimmer Biomet	49	5.76	7.82	11.88	12.67
POD2	DePuy Synthes	9	6.44	9.15	9.75	9.94
POD2	Implantcast	5	4.84	7.95	8.56	8.77
POD3	Exactech	2	8.38	8.93	9.27	9.38
POD3	Zimmer Biomet	32	4.62	6.39	7.41	9.15
POD3	DePuy Synthes	0				
POD3	Implantcast	0				
POD5	Exactech	105	3.04	4.82	8.22	10.18
POD5	Zimmer Biomet	59	3.49	6.33	8.41	10.55
POD5	DePuy Synthes	6	2.91	3.69	4.54	4.95
POD5	Implantcast	4	1.25	2.20	3.54	3.99
POD7	Exactech	6	3.19	4.65	7.70	9.01
POD7	Zimmer Biomet	5	2.65	7.14	9.44	10.21
POD7	DePuy Synthes	1	3.70	3.70	3.70	3.70

POD7	Implantcast	0				
POD14	Exactech	99	0.26	0.49	1.13	1.58
POD14	Zimmer Biomet	72	0.23	0.48	0.96	1.42
POD14	DePuy Synthes	8	0.29	0.64	2.69	4.35
POD14	Implantcast	4	0.15	0.26	0.39	0.43

ESR by Implant company

Timepoint	Group	n	median	p75	p90	p95
Pre-op	Exactech	136	11.00	18.25	36.00	64.00
Pre-op	Zimmer Biomet	96	10.00	18.50	28.50	33.25
Pre-op	DePuy Synthes	12	15.00	19.75	22.00	28.75
Pre-op	Implantcast	6	12.00	13.50	18.50	20.75
POD1	Exactech	1	23.00	23.00	23.00	23.00
POD1	Zimmer Biomet	0				
POD1	DePuy Synthes	1	2.00	2.00	2.00	2.00
POD1	Implantcast	0				
POD2	Exactech	93	6.00	13.00	20.80	27.40
POD2	Zimmer Biomet	47	3.00	7.50	15.40	19.40
POD2	DePuy Synthes	8	3.50	10.75	20.20	21.60
POD2	Implantcast	5	2.00	2.00	4.40	5.20
POD3	Exactech	4	17.50	21.50	24.20	25.10
POD3	Zimmer Biomet	33	16.00	29.00	34.80	50.80
POD3	DePuy Synthes	0				
POD3	Implantcast	0				
POD5	Exactech	105	23.00	37.00	46.00	50.80
POD5	Zimmer Biomet	60	23.50	35.00	50.60	60.05
POD5	DePuy Synthes	5	16.00	20.00	26.00	28.00
POD5	Implantcast	4	5.00	8.50	9.40	9.70
POD7	Exactech	5	32.00	39.00	41.40	42.20
POD7	Zimmer Biomet	5	24.00	49.00	60.40	64.20
POD7	DePuy Synthes	1	38.00	38.00	38.00	38.00
POD7	Implantcast	0				
POD14	Exactech	99	23.00	33.00	46.00	48.00
POD14	Zimmer Biomet	71	19.00	36.00	51.00	61.50
POD14	DePuy Synthes	7	14.00	22.00	29.80	30.40
POD14	Implantcast	4	11.00	21.00	24.60	25.80

Supplementary Table S4. Age-Stratified Percentile Trajectories (<70 vs >=70 years)

Per-timepoint medians, 75th, 90th, and 95th percentiles (with per-stratum n) for CRP (mg/dL) and ESR (mm/h). Strata with fewer than five observations at a timepoint are degenerate and are shown for completeness only, not as estimates; this includes the POD 1 ESR window (n = 2), reported as not estimable in Table 3.

CRP by Age group

Timepoint	Group	n	median	p75	p90	p95

Pre-op	<70	22	0.09	0.22	0.40	0.61
Pre-op	>=70	247	0.09	0.29	0.94	2.40
POD1	<70	2	0.61	0.66	0.68	0.69
POD1	>=70	19	2.28	3.15	4.15	4.30
POD2	<70	11	6.35	7.75	9.30	13.37
POD2	>=70	160	6.25	8.79	10.45	13.02
POD3	<70	5	1.53	1.65	3.59	4.24
POD3	>=70	29	5.41	7.11	8.69	9.77
POD5	<70	16	2.04	4.19	6.53	6.73
POD5	>=70	158	3.13	5.08	8.46	10.34
POD7	<70	0				
POD7	>=70	12	3.19	5.59	10.00	10.62
POD14	<70	15	0.22	0.40	0.45	0.45
POD14	>=70	168	0.25	0.54	1.18	1.81

ESR by Age group

Timepoint	Group	n	median	p75	p90	p95
Pre-op	<70	21	9.00	15.00	24.00	28.00
Pre-op	>=70	229	11.00	19.00	31.00	44.60
POD1	<70	0				
POD1	>=70	2	12.50	17.75	20.90	21.95
POD2	<70	11	3.00	4.50	14.00	17.50
POD2	>=70	142	5.00	11.00	20.00	24.90
POD3	<70	5	17.00	20.00	23.60	24.80
POD3	>=70	32	15.50	29.00	34.90	51.65
POD5	<70	17	18.00	28.00	38.60	41.20
POD5	>=70	157	22.00	36.00	46.40	56.20
POD7	<70	0				
POD7	>=70	11	32.00	41.00	49.00	58.50
POD14	<70	15	12.00	22.00	34.60	39.70
POD14	>=70	166	23.00	33.00	48.00	56.00

Supplementary Table S5. Sex-Stratified Percentile Trajectories

Per-timepoint medians, 75th, 90th, and 95th percentiles (with per-stratum n) for CRP (mg/dL) and ESR (mm/h). Strata with fewer than five observations at a timepoint are degenerate and are shown for completeness only, not as estimates; this includes the POD 1 ESR window (n = 2), reported as not estimable in Table 3.

CRP by Sex

Timepoint	Group	n	median	p75	p90	p95
Pre-op	F	173	0.09	0.27	0.94	2.52
Pre-op	M	96	0.11	0.29	0.76	1.87
POD1	F	14	2.29	3.21	4.05	4.17
POD1	M	7	2.22	2.65	3.72	4.25
POD2	F	113	6.25	7.95	10.39	12.83
POD2	M	58	6.40	8.85	10.31	13.09

POD3	F	22	5.15	6.88	8.38	9.44
POD3	M	12	4.07	6.96	7.38	13.11
POD5	F	116	2.96	4.87	7.45	9.32
POD5	M	58	3.52	6.41	9.79	12.97
POD7	F	8	3.19	4.04	5.69	6.42
POD7	M	4	6.49	10.48	10.78	10.88
POD14	F	120	0.21	0.45	0.92	1.20
POD14	M	63	0.36	0.55	1.51	2.51

ESR by Sex

Timepoint	Group	n	median	p75	p90	p95
Pre-op	F	166	12.00	20.75	36.00	52.25
Pre-op	M	84	9.00	17.00	21.70	28.85
POD1	F	2	12.50	17.75	20.90	21.95
POD1	M	0				
POD2	F	100	5.00	12.25	21.00	26.05
POD2	M	53	4.00	7.00	15.00	17.60
POD3	F	24	19.50	26.00	32.80	42.50
POD3	M	13	12.00	29.00	33.80	46.20
POD5	F	117	21.00	34.00	44.40	48.40
POD5	M	57	27.00	40.00	53.40	59.20
POD7	F	7	32.00	38.50	50.60	59.30
POD7	M	4	33.50	44.50	47.20	48.10
POD14	F	118	23.00	32.75	43.30	50.15
POD14	M	63	16.00	33.50	49.60	58.70

Supplementary Table S6. LMM Convergence Diagnostics for 8 Prespecified Interaction Tests

Per-test optimizer used, convergence flag, Hessian singularity status, log-likelihoods of the full and reduced models, LRT statistic, degrees of freedom, p-value, and reason for non-estimability (NE). Six of eight tests reflected a singular Hessian of the full interaction model under all four optimizers attempted (L-BFGS-B, Powell, BFGS, and Nelder–Mead). The df column is the nominal (k-1)(T-1); for non-estimable rows the realized residual df used by the model fit may be one lower (e.g., 17, 5, and 11 rather than 18, 6, and 12) when a level x timepoint cell is empty, which does not affect the non-estimable designation. For the two estimable rows the nominal and realized df coincide (diagnosis x CRP df = 12; sex x CRP df = 6). Stratum sizes in the NE-reason column are given as procedures/observations (Implantcast 5 procedures/20 observations); procedure counts match Table 1.

subgroup_factor	biomarker	levels	optimizer	converged	hessian_singular	llf_full	llf_reduced	lrt_statistic	df	p_value	ne_reason
Diagnosis_group	CRP	CTA/Massive RCT/Primary OA	powell	True	False	-958.4	-966.3	15.791	12	0.201	
Diagnosis_group	ESR	CTA/Massive RCT/Primary OA	none	False	True		-3145.9	NE	12	NE	full-model

											optimizer failed (singular Hessian)
Implant_company	CRP	DePuy Synthes/Exactech/Implantcast/Zimmer Biomet	none	False	True		-989.7	NE	18	NE	full-model optimizer failed (singular Hessian); sparse stratum: Implantcast 5 procedures/20 observations
Implant_company	ESR	DePuy Synthes/Exactech/Implantcast/Zimmer Biomet	none	False	True		-3190.7	NE	18	NE	full-model optimizer failed (singular Hessian); sparse stratum: Implantcast 5 procedures/19 observations
Age_group	CRP	<70/>=70	none	False	True		-989.1	NE	6	NE	full-model optimizer failed (singular Hessian)
Age_group	ESR	<70/>=70	none	False	True		-3190.8	NE	6	NE	full-model optimizer failed (singular Hessian)
Sex	CRP	F/M	powell	True	False	-984.	-988.4	8.079	6	0.2324	

							4					
Sex	ESR	F/M		none	False	True		-3192.3	NE	6	NE	full-model optimizer failed (singular Hessian)

Supplementary Table S7. Non-Parametric Bootstrap 95% Confidence Intervals for Empirical Percentile Reference Values

Non-parametric bootstrap with 2,000 replicates (fixed random seed); interval bounds are the 2.5th and 97.5th percentiles of the bootstrap distribution. Per-timepoint denominators replicate the closest-to-nominal-day deduplication used for Tables 2 and 3. Timepoints with n < 5 are not estimable for bootstrap intervals (NE). Units: mg/dL for CRP, mm/h for ESR.

marker	timepoint	n	p50	p50_CI_low	p50_CI_high	p75	p75_CI_low	p75_CI_high	p90	p90_CI_low	p90_CI_high	p95	p95_CI_low	p95_CI_high
CRP	Pre-op	269	0.09	0.08	0.11	0.29	0.21	0.4	0.93	0.572	1.96	2.34	1.632	3.24
CRP	POD 1	21	2.22	0.85	3.02	3.04	2.28	4.13	4.13	3.02	4.78	4.25	3.27	4.78
CRP	POD 2	171	6.25	5.76	6.59	8.79	7.475	9.07	10.45	9.31	12.07	13.33	10.65	14.58
CRP	POD 3	34	4.77	2.99	6.06	7.04	5.41	7.402	8.17	7.053	9.95	9.65	7.312	20.08
CRP	POD 5	174	3.06	2.62	3.655	5.08	4.49	6.29	8.24	7.214	10.06	10.23	8.592	12.31
CRP	POD 7	12	3.19	1.215	6.105	5.59	3.0	10.485	10.00	3.669	10.98	10.62	5.07	10.98
CRP	POD 14	183	0.25	0.2	0.32	0.48	0.41	0.6	1.11	0.854	1.42	1.53	1.183	2.534
ESR	Pre-op	250	11.00	10.0	13.0	18.75	17.0	21.0	30.10	25.1	37.2	41.00	33.65	65.1
ESR	POD 1	2		NE	NE		NE	NE		NE	NE		NE	NE
ESR	POD 2	153	5.00	3.0	6.0	11.00	8.0	14.0	20.00	15.0	22.61	23.80	20.0	28.0
ESR	POD 3	37	16.00	12.0	21.0	26.00	20.0	30.0	34.40	27.2	61.0	47.40	29.2	63.0

ESR	POD 5	174	21.00	18.5	27.0	36.00	31.75	40.0	46.00	42.0	51.4	55.35	46.7	60.0
ESR	POD 7	11	32.00	13.0	43.0	41.00	28.0	58.5	49.00	38.0	68.0	58.50	39.0	68.0
ESR	POD 14	181	22.00	18.0	23.0	33.00	29.0	37.0	47.00	42.0	51.0	56.00	48.0	62.0

Supplementary Table S8. Per-Procedure Implant Extraction Audit (De-identified)

De-identified per-procedure record of the implant-component extraction audit (analytic cohort N = 214), keyed solely by procedure number (no patient identifiers). Manufacturer, product line, and fixation classification were transcribed from intraoperative implant sticker photographs by five independent reviewers (each visually inspecting ~44 of the 218 source images) using vendor-specific label nomenclature, with discordances reconciled by author review of the sticker image. Most images yielded a high-confidence reading, with 13 graded medium-confidence and 2 low-confidence. Two humeral stem labels that were absent or illegible on the sticker image (Nos 183 and 196) were resolved from the surgeon-maintained operative record, and one indeterminate humeral-fixation label (No 104) was assigned to the cementless stratum per the institutional default (Audit note column). Bone-cement labels co-existing with a press-fit humeral stem label in approximately ten procedures were adjudicated to ancillary use rather than humeral fixation.

No	Vendor	Product	Humeral fixation	Baseplate fixation	Augmented baseplate	Constrained liner	Audit note
1	Zimmer Biomet	Zimmer Trabecular Metal	cementless	cementless			
2	Zimmer Biomet	Zimmer Trabecular Metal	cementless	cementless			
3	Exactech	Equinoxe Reverse Shoulder	cementless	cementless			
4	Exactech	Equinoxe Reverse Shoulder	cementless	cementless			
5	Exactech	Equinoxe Reverse Shoulder	cementless	cementless			
6	Zimmer Biomet	Zimmer Trabecular Metal	cementless	cementless			
7	Zimmer Biomet	Zimmer Trabecular Metal	cementless	cementless			
8	Exactech	Equinoxe Reverse Shoulder	cementless	cementless			

9	Exactech	Equinoxe Reverse Shoulder	cementless	cementless
10	Exactech	Equinoxe Reverse Shoulder	cementless	cementless
11	Implantcast	Agilon	cementless	cementless
12	Exactech	Equinoxe Reverse Shoulder	cementless	cementless
13	Implantcast	Agilon	cemented	cementless
14	Exactech	Equinoxe Reverse Shoulder	cementless	cementless
15	Exactech	Equinoxe Reverse Shoulder	cementless	cementless
16	DePuy Synthes	Delta Xtend	cemented	cementless
17	Exactech	Equinoxe Reverse Shoulder	cementless	cementless
18	Exactech	Equinoxe Reverse Shoulder	cementless	cementless
19	Exactech	Equinoxe Reverse Shoulder	cementless	cementless
20	Exactech	Equinoxe Reverse Shoulder	cementless	cementless
21	Exactech	Equinoxe Reverse Shoulder	cementless	cementless
22	DePuy Synthes	Delta Xtend	cemented	cementless
23	Implantcast	Agilon	cementless	cementless
24	Exactech	Equinoxe Reverse Shoulder	cementless	cementless
25	Implantcast	Agilon	cementless	cementless
26	Exactech	Equinoxe Reverse Shoulder	cementless	cementless
27	Exactech	Equinoxe Reverse Shoulder	cementless	cementless
28	Exactech	Equinoxe Reverse Shoulder	cementless	cementless
29	DePuy Synthes	Delta Xtend	cemented	cementless
30	Exactech	Equinoxe	cementless	cementless

		Reverse Shoulder				
31	Exactech	Equinoxe Reverse Shoulder	cementless	cementless		
32	Exactech	Equinoxe Reverse Shoulder	cementless	cementless		
33	Implantcast	Agilon	cementless	cementless		
34	Exactech	Equinoxe Reverse Shoulder	cementless	cementless		
35	Exactech	Equinoxe Reverse Shoulder	cementless	cementless		
36	Exactech	Equinoxe Reverse Shoulder	cementless	cementless		
37	DePuy Synthes	Delta Xtend	cementless	cementless		
38	Zimmer Biomet	Zimmer Trabecular Metal	cementless	cementless		
39	Zimmer Biomet	Zimmer Trabecular Metal	cementless	cementless		
40	Exactech	Equinoxe Reverse Shoulder	cementless	cementless		
41	Exactech	Equinoxe Reverse Shoulder	cementless	cementless		
42	Zimmer Biomet	Zimmer Trabecular Metal	cementless	cementless		
43	Exactech	Equinoxe Reverse Shoulder	cementless	cementless		Yes
44	Exactech	Equinoxe Reverse Shoulder	cementless	cementless		
45	Exactech	Equinoxe Reverse Shoulder	cementless	cementless	Yes	
46	Zimmer Biomet	Zimmer Trabecular Metal	cementless	cementless		
47	Exactech	Equinoxe Reverse Shoulder	cementless	cementless		
48	Zimmer Biomet	Zimmer Trabecular Metal	cementless	cementless		

49	DePuy Synthes	Delta Xtend	cemented	cementless	
50	Exactech	Equinoxe Reverse Shoulder	cementless	cementless	
52	Zimmer Biomet	Zimmer Trabecular Metal	cementless	cementless	
53	Exactech	Equinoxe Reverse Shoulder	cementless	cementless	
54	Zimmer Biomet	Zimmer Trabecular Metal	cementless	cementless	
55	Exactech	Equinoxe Reverse Shoulder	cementless	cementless	
56	DePuy Synthes	Delta Xtend	cemented	cementless	
57	Exactech	Equinoxe Reverse Shoulder	cementless	cementless	
58	DePuy Synthes	Delta Xtend	cemented	cementless	
59	Zimmer Biomet	Zimmer Trabecular Metal	cementless	cementless	
60	DePuy Synthes	Delta Xtend	cemented	cementless	
61	DePuy Synthes	Delta Xtend	cemented	cementless	
62	Exactech	Equinoxe Reverse Shoulder	cementless	cementless	
63	Zimmer Biomet	Zimmer Trabecular Metal	cementless	cementless	
64	Zimmer Biomet	Zimmer Trabecular Metal	cementless	cementless	
65	Exactech	Equinoxe Reverse Shoulder	cementless	cementless	
66	Exactech	Equinoxe Reverse Shoulder	cementless	cementless	
67	Zimmer Biomet	Zimmer Trabecular Metal	cementless	cementless	
68	Zimmer Biomet	Zimmer Trabecular Metal	cementless	cementless	
69	Zimmer Biomet	Zimmer Trabecular Metal	cementless	cementless	
70	Exactech	Equinoxe	cementless	cementless	Yes

		Reverse Shoulder			
72	Exactech	Equinoxe Reverse Shoulder	cementless	cementless	
73	Exactech	Equinoxe Reverse Shoulder	cementless	cementless	
74	Exactech	Equinoxe Reverse Shoulder	cementless	cementless	
75	Zimmer Biomet	Zimmer Trabecular Metal	cementless	cementless	
76	Exactech	Equinoxe Reverse Shoulder	cementless	cementless	
77	Exactech	Equinoxe Reverse Shoulder	cementless	cementless	
78	Exactech	Equinoxe Reverse Shoulder	cementless	cementless	
79	Exactech	Equinoxe Reverse Shoulder	cementless	cementless	
80	Exactech	Equinoxe Reverse Shoulder	cementless	cementless	
81	Exactech	Equinoxe Reverse Shoulder	cementless	cementless	
82	Exactech	Equinoxe Reverse Shoulder	cementless	cementless	
83	Exactech	Equinoxe Reverse Shoulder	cementless	cementless	
84	Exactech	Equinoxe Reverse Shoulder	cementless	cementless	Yes
85	Exactech	Equinoxe Reverse Shoulder	cementless	cementless	
86	Exactech	Equinoxe Reverse Shoulder	cementless	cementless	
87	Exactech	Equinoxe Reverse Shoulder	cementless	cementless	
88	Exactech	Equinoxe Reverse Shoulder	cementless	cementless	

90	Exactech	Equinoxe Reverse Shoulder	cementless	cementless			
91	Exactech	Equinoxe Reverse Shoulder	cementless	cementless			
92	Exactech	Equinoxe Reverse Shoulder	cementless	cementless			
93	Exactech	Equinoxe Reverse Shoulder	cementless	cementless			
94	Exactech	Equinoxe Reverse Shoulder	cementless	cementless			
95	Exactech	Equinoxe Reverse Shoulder	cementless	cementless			
96	Exactech	Equinoxe Reverse Shoulder	cementless	cementless			
97	Exactech	Equinoxe Reverse Shoulder	cementless	cementless			
98	Exactech	Equinoxe Reverse Shoulder	cementless	cementless			
99	Exactech	Equinoxe Reverse Shoulder	cementless	cementless	Yes		
100	Exactech	Equinoxe Reverse Shoulder	cementless	cementless			
101	Exactech	Equinoxe Reverse Shoulder	cementless	cementless			
102	Exactech	Equinoxe Reverse Shoulder	cementless	cementless			
103	Exactech	Equinoxe Reverse Shoulder	cementless	cementless			
104	Exactech	Equinoxe Reverse Shoulder	cementless	cementless			Humeral fixation assigned by institutional default (indeterminate sticker label)
105	Exactech	Equinoxe Reverse Shoulder	cementless	cementless			
106	Exactech	Equinoxe Reverse	cementless	cementless			

		Shoulder		
107	Exactech	Equinoxe Reverse Shoulder	cementless	cementless
108	Exactech	Equinoxe Reverse Shoulder	cementless	cementless
109	Exactech	Equinoxe Reverse Shoulder	cementless	cementless
110	Exactech	Equinoxe Reverse Shoulder	cementless	cementless
111	Exactech	Equinoxe Reverse Shoulder	cementless	cementless
112	Exactech	Equinoxe Reverse Shoulder	cementless	cementless
113	Exactech	Equinoxe Reverse Shoulder	cementless	cementless
114	Exactech	Equinoxe Reverse Shoulder	cementless	cementless
115	Exactech	Equinoxe Reverse Shoulder	cementless	cementless
116	Exactech	Equinoxe Reverse Shoulder	cementless	cementless
117	Exactech	Equinoxe Reverse Shoulder	cementless	cementless
118	Exactech	Equinoxe Reverse Shoulder	cementless	cementless
119	Exactech	Equinoxe Reverse Shoulder	cementless	cementless
120	Exactech	Equinoxe Reverse Shoulder	cementless	cementless
121	Exactech	Equinoxe Reverse Shoulder	cementless	cementless
122	Exactech	Equinoxe Reverse Shoulder	cementless	cementless
123	Exactech	Equinoxe Reverse Shoulder	cementless	cementless
124	Exactech	Equinoxe	cementless	cementless

		Reverse Shoulder		
125	Exactech	Equinoxe Reverse Shoulder	cementless	cementless
126	Exactech	Equinoxe Reverse Shoulder	cementless	cementless
127	Exactech	Equinoxe Reverse Shoulder	cementless	cementless
128	Exactech	Equinoxe Reverse Shoulder	cementless	cementless
129	Exactech	Equinoxe Reverse Shoulder	cementless	cementless
130	Exactech	Equinoxe Reverse Shoulder	cementless	cementless
131	Exactech	Equinoxe Reverse Shoulder	cementless	cementless
132	Exactech	Equinoxe Reverse Shoulder	cementless	cementless
133	Exactech	Equinoxe Reverse Shoulder	cementless	cementless
134	Exactech	Equinoxe Reverse Shoulder	cementless	cementless
135	Exactech	Equinoxe Reverse Shoulder	cementless	cementless
136	Exactech	Equinoxe Reverse Shoulder	cementless	cementless
137	Exactech	Equinoxe Reverse Shoulder	cementless	cementless
138	Zimmer Biomet	Comprehensive	cementless	cementless
139	Exactech	Equinoxe Reverse Shoulder	cementless	cementless
140	Zimmer Biomet	Comprehensive	cementless	cementless
141	Exactech	Equinoxe Reverse Shoulder	cementless	cementless
142	Zimmer Biomet	Comprehensive	cementless	cementless
143	Exactech	Equinoxe Reverse	cementless	cementless

		Shoulder		
144	Zimmer Biomet	Comprehensive	cementless	cementless
145	Exactech	Equinoxe Reverse Shoulder	cementless	cementless
146	Zimmer Biomet	Comprehensive	cementless	cementless
147	Exactech	Equinoxe Reverse Shoulder	cementless	cementless
148	Exactech	Equinoxe Reverse Shoulder	cementless	cementless
149	Exactech	Equinoxe Reverse Shoulder	cementless	cementless
150	Exactech	Equinoxe Reverse Shoulder	cementless	cementless
151	Zimmer Biomet	Comprehensive	cementless	cementless
152	Zimmer Biomet	Comprehensive	cementless	cementless
153	Zimmer Biomet	Comprehensive	cementless	cementless
154	Exactech	Equinoxe Reverse Shoulder	cementless	cementless
155	Zimmer Biomet	Comprehensive	cementless	cementless
156	Zimmer Biomet	Comprehensive	cementless	cementless
157	Exactech	Equinoxe Reverse Shoulder	cementless	cementless
158	Zimmer Biomet	Comprehensive	cementless	cementless
160	Exactech	Equinoxe Reverse Shoulder	cementless	cementless
161	Zimmer Biomet	Comprehensive	cementless	cementless
162	Exactech	Equinoxe Reverse Shoulder	cementless	cementless
163	Zimmer Biomet	Comprehensive	cementless	cementless
164	Zimmer Biomet	Comprehensive	cementless	cementless
165	Zimmer Biomet	Comprehensive	cementless	cementless
166	Zimmer Biomet	Comprehensive	cementless	cementless
167	Zimmer Biomet	Comprehensive	cementless	cementless
168	Zimmer Biomet	Comprehensive	cementless	cementless
169	Zimmer Biomet	Comprehensive	cementless	cementless
170	Zimmer Biomet	Comprehensive	cementless	cementless

171	Zimmer Biomet	Comprehensive	cementless	cementless	
172	Zimmer Biomet	Comprehensive	cementless	cementless	
173	Zimmer Biomet	Comprehensive	cementless	cementless	
174	Zimmer Biomet	Comprehensive	cementless	cementless	
175	Zimmer Biomet	Comprehensive	cementless	cementless	
176	Zimmer Biomet	Comprehensive	cementless	cementless	
177	Zimmer Biomet	Comprehensive	cementless	cementless	
178	Zimmer Biomet	Comprehensive	cementless	cementless	
179	Zimmer Biomet	Comprehensive	cementless	cementless	
180	Zimmer Biomet	Comprehensive	cementless	cementless	
181	Zimmer Biomet	Comprehensive	cementless	cementless	
182	Zimmer Biomet	Comprehensive	cementless	cementless	
183	Zimmer Biomet	Comprehensive	cementless	cementless	Humeral stem data substituted from operative record (no sticker image)
184	Zimmer Biomet	Comprehensive	cementless	cementless	
185	Zimmer Biomet	Comprehensive	cementless	cementless	
186	Zimmer Biomet	Comprehensive	cementless	cementless	
187	Zimmer Biomet	Comprehensive	cementless	cementless	
188	Zimmer Biomet	Comprehensive	cementless	cementless	
189	Zimmer Biomet	Comprehensive	cementless	cementless	
190	Zimmer Biomet	Comprehensive	cementless	cementless	
191	Zimmer Biomet	Comprehensive	cementless	cementless	
192	Zimmer Biomet	Comprehensive	cementless	cementless	
193	Zimmer Biomet	Comprehensive	cementless	cementless	
194	Zimmer Biomet	Comprehensive	cementless	cementless	
195	Zimmer Biomet	Comprehensive	cementless	cementless	
196	Zimmer Biomet	Zimmer Trabecular Metal	cementless	cementless	Humeral stem data substituted from operative record (stem label absent from sheet)
197	Zimmer Biomet	Comprehensive	cementless	cementless	
198	Zimmer Biomet	Comprehensive	cementless	cementless	
199	Zimmer Biomet	Comprehensive	cementless	cementless	
200	Exactech	Equinoxe Reverse Shoulder	cementless	cementless	

201	Zimmer Biomet	Comprehensive	cementless	cementless	
202	Zimmer Biomet	Comprehensive	cementless	cementless	
203	Zimmer Biomet	Comprehensive	cementless	cementless	
204	Zimmer Biomet	Comprehensive	cementless	cementless	
205	Zimmer Biomet	Comprehensive	cementless	cementless	
206	Exactech	Equinoxe Reverse Shoulder	cementless	cementless	Yes
207	Zimmer Biomet	Comprehensive	cementless	cementless	
208	Zimmer Biomet	Comprehensive	cementless	cementless	
209	Zimmer Biomet	Comprehensive	cementless	cementless	
210	Zimmer Biomet	Comprehensive	cementless	cementless	
211	Zimmer Biomet	Comprehensive	cementless	cementless	
212	Zimmer Biomet	Comprehensive	cementless	cementless	
213	Zimmer Biomet	Comprehensive	cementless	cementless	
214	Zimmer Biomet	Comprehensive	cementless	cementless	
215	Zimmer Biomet	Comprehensive	cementless	cementless	
216	Zimmer Biomet	Comprehensive	cementless	cementless	
217	Zimmer Biomet	Comprehensive	cementless	cementless	
218	Zimmer Biomet	Comprehensive	cementless	cementless	

Supplementary Table S9. Timepoint Denominator Audit

Per-window accounting of the analytic denominator against the full cohort of 214 procedures. Categories are mutually exclusive and assigned in priority order: "recovered in adjacent window" denotes a protocolized draw that fell into a neighboring window (POD 2 with POD 1 or POD 3; POD 5 with POD 3 or POD 7; POD 14 with POD 7), so the measurement still contributes to the reference curve rather than being lost — this category is defined for the routine windows only, the non-routine windows having no protocolized draw that could drift; "blood drawn, assay not ordered" denotes a recorded venipuncture within the window whose panel did not include that marker; "no blood sampling in window" denotes no venipuncture within the window at all, and at the routine windows procedures in this category additionally had no assay in either adjacent window, adjacent recovery being assessed first. Pooled across POD 1 to POD 3, CRP was available in 205 of 214 procedures (95.8%) and ESR in 185 of 214 (86.4%); at least one postoperative CRP was available in 214 of 214 and at least one postoperative ESR in 212 of 214.

Marker	Timepoint	Window	n analyzed (%)	Not analyzed	Recovered in adjacent window	Blood drawn, assay not ordered	No blood sampling in window
CRP	POD 1	Non-routine	21 (9.8%)	193	0	193	0
CRP	POD 2	Routine	171 (79.9%)	43	34	0	9
CRP	POD 3	Non-routine	34 (15.9%)	180	0	11	169
CRP	POD 5	Routine	174 (81.3%)	40	7	5	28

CRP	POD 7	Non-routine	12 (5.6%)	202	0	3	199
CRP	POD 14	Routine	183 (85.5%)	31	4	0	27
ESR	POD 1	Non-routine	2 (0.9%)	212	0	212	0
ESR	POD 2	Routine	153 (71.5%)	61	32	19	10
ESR	POD 3	Non-routine	37 (17.3%)	177	0	8	169
ESR	POD 5	Routine	174 (81.3%)	40	9	4	27
ESR	POD 7	Non-routine	11 (5.1%)	203	0	4	199
ESR	POD 14	Routine	181 (84.6%)	33	4	2	27

Supplementary Table S10. Sensitivity Analysis for Informative Missingness at Postoperative Day 14

Comparison of baseline, perioperative, and early postoperative characteristics between procedures that contributed a POD 14 CRP measurement (n = 183) and those that did not (n = 31), performed post hoc at revision to assess whether the available-case reference curve is subject to informative missingness. The grouping is defined by the CRP draw; the POD 14 ESR denominator is 181, the panel omitting ESR in two procedures (Table S9). SMD denotes the standardized mean difference, for which an absolute value below 0.10 conventionally indicates negligible imbalance. Continuous variables are median [interquartile range] unless otherwise stated; categorical variables are n (%); postoperative CRP values are the closest-to-nominal-day draw, as in Tables 2 and 3. Postoperative day of discharge is shown both as mean ± SD and as median [IQR] because the median is degenerate at the protocolized discharge day. All comparisons are available-case; variables with incomplete data (sampled / not sampled) were body mass index 180/30, postoperative day of discharge 182/31, preoperative ESR 182/31, POD 2 CRP 145/26, and POD 5 CRP 147/27, and all other variables were complete in both groups.

Variable	POD 14 draw obtained (n = 183)	No POD 14 draw (n = 31)	SMD	p	Test
Age (years)	78.2 ± 6.2	79.5 ± 6.9	-0.20	0.319	Mann–Whitney U
BMI (kg/m²)	24.8 [22.7–27.1]	24.5 [22.6–28.8]	-0.14	0.805	Mann–Whitney U
Anesthesia duration (min)	130.0 [120.0–140.0]	125.0 [120.0–137.5]	-0.01	0.504	Mann–Whitney U
Postoperative day of discharge, mean ± SD	5.3 ± 2.1	6.8 ± 4.7	-0.40	<0.001	Mann–Whitney U
Postoperative day of discharge, median [IQR]	5.0 [5.0–5.0]	5.0 [5.0–6.5]	-0.40	<0.001	Mann–Whitney U
Preoperative CRP (mg/dL)	0.1 [0.1–0.2]	0.1 [0.0–0.1]	-0.07	0.231	Mann–Whitney U
Preoperative ESR (mm/h)	9.0 [5.0–17.0]	12.0 [4.0–16.0]	-0.14	0.759	Mann–Whitney U
POD 2 CRP (mg/dL)	6.1 [4.3–8.3]	7.3 [5.2–8.9]	-0.20	0.271	Mann–Whitney U
POD 5 CRP (mg/dL)	3.1 [1.7–5.1]	2.6 [0.8–4.5]	0.09	0.242	Mann–Whitney U
Female sex	120 (65.6%)	21 (67.7%)	-0.05	0.814	Chi-square

ASA ≥ 3	94 (51.4%)	18 (58.1%)	-0.14	0.490	Chi-square
Diagnosis — cuff tear arthropathy	95 (51.9%)	20 (64.5%)	-0.26	0.193	Chi-square
Diabetes mellitus	50 (27.3%)	9 (29.0%)	-0.04	0.844	Chi-square
Hypertension	120 (65.6%)	20 (64.5%)	0.02	0.909	Chi-square
Chronic kidney disease	7 (3.8%)	2 (6.5%)	-0.12	0.621	Fisher's exact
Current or former smoker	16 (8.7%)	0 (0.0%)	0.44	0.136	Fisher's exact
Implant — Exactech	99 (54.1%)	17 (54.8%)	-0.01	0.939	Chi-square
Cemented humeral stem	8 (4.4%)	1 (3.2%)	0.06	1.000	Fisher's exact

Supplementary Table S11. Within-Patient Fold Change and Velocity Relative to the Individual Preoperative Baseline

Within-patient change from each patient's own last preoperative measurement. Fold change is the postoperative value divided by the preoperative value; delta is the postoperative minus the preoperative value (mg/dL for CRP, mm/h for ESR); velocity is delta divided by the nominal postoperative day. Only procedures contributing a paired preoperative and timepoint value are included. Because the median preoperative CRP (0.08 mg/dL) lies close to the assay reporting floor, CRP ratios have a near-zero denominator and are numerically unstable; the absolute change is therefore reported alongside every ratio, and for CRP it is the more stable reference quantity. Baseline values were available for CRP in 214 of 214 procedures and for ESR in 213 of 214. As in Table 3, the POD 1 ESR window rests on two paired observations; its summaries are degenerate and are shown for completeness only, not as estimates.

Marker	Timepoint	Window	n	Fold change, median [IQR]	Fold change, 90th	Fold change, 95th	Absolute change, median [IQR]	Velocity, median per day	At or below own baseline (%)
CRP	POD 1	Non-routine	21	15.10 [10.40–28.33]	31.71	32.70	2.10 [0.65–2.85]	2.10	0.0
CRP	POD 2	Routine	171	63.83 [24.30–127.31]	233.50	303.00	5.97 [3.96–8.18]	2.98	0.0
CRP	POD 3	Non-routine	34	38.35 [24.11–86.42]	270.00	336.68	4.71 [2.29–6.72]	1.57	0.0
CRP	POD 5	Routine	174	29.30 [12.43–63.62]	130.15	210.79	2.80 [1.44–4.75]	0.56	2.9
CRP	POD 7	Non-routine	12	24.19 [7.72–65.14]	238.35	294.22	2.79 [1.16–5.56]	0.40	8.3
CRP	POD 14	Routine	183	2.57 [1.05–6.00]	12.65	17.98	0.11 [0.00–0.35]	0.01	25.1
ESR	POD 1	Non-routine	2	1.01 [0.56–	1.74	1.83	-3.00 [-	-3.00	50.0

				1.46]			10.00 to 4.00]		
ESR	POD 2	Routine	153	0.60 [0.29–1.00]	1.73	2.33	-2.00 [-9.00 to 0.00]	-1.00	77.8
ESR	POD 3	Non-routine	37	1.73 [1.00–2.60]	4.27	6.00	7.00 [0.00–11.00]	2.33	27.0
ESR	POD 5	Routine	174	1.78 [0.91–3.74]	5.97	8.25	9.50 [-1.00 to 22.00]	1.90	32.2
ESR	POD 7	Non-routine	11	3.25 [1.50–6.02]	8.00	12.00	21.00 [4.50–32.50]	3.00	27.3
ESR	POD 14	Routine	181	2.00 [1.15–3.29]	5.25	7.67	9.00 [2.00–19.00]	0.64	21.0
