# Supplementary material for: Timepoint-Specific Percentile Reference Curves for C-Reactive Protein and Erythrocyte Sedimentation Rate After Uncomplicated Primary Reverse Shoulder Arthroplasty: A Single-Center Cohort Study
Source: Diagnostics (Basel). 2026 Sep 16;16(18):2993. doi: 10.3390/diagnostics16182993 (PMC13605674; doi:10.3390/diagnostics16182993)
Supplement: Supplementary file 1 [file diagnostics-16-02993-s001.zip › Table S12. STROBE_checklist_REV1_260822.pdf]

Table S12. Manuscript: Timepoint-Specific Percentile Reference Curves for C-Reactive Protein and Erythrocyte Sedimentation Rate After Uncomplicated Primary Reverse Shoulder Arthroplasty: A Single-Center Cohort Study. Revision 1 (manuscript ID diagnostics-4405622). Items updated at this revision: 6(a) adds the rationale for restricting the cohort to elective degenerative indications; 7 adds the new subsection Definition of "Uncomplicated"; 10 reflects the rewritten Sample Size section; 12(c) and 12(e) reflect the analyses added in response to the reviewers (Supplementary Tables S9–S11); and 14(b) states the per-variable completeness of Table 1.

Section/Topic	Item No.	Recommendation	Reported on page/section
Title and abstract	1(a)	Indicate the study's design with a commonly used term in the title or the abstract.	Title ("A Single-Center Cohort Study"); Abstract – Methods; Materials and Methods – Study Design and Setting
	1(b)	Provide in the abstract an informative and balanced summary of what was done and what was found.	Abstract (structured)
Introduction — Background/rationale	2	Explain the scientific background and rationale for the investigation being reported.	Introduction, paras 1–2
Objectives	3	State specific objectives, including any pre-specified hypotheses.	Introduction, para 3
Methods — Study design	4	Present key elements of study design early in the paper.	Materials and Methods – Study Design and Setting
Setting	5	Describe the setting, locations, and relevant dates, including periods of recruitment, exposure, follow-up, and data collection.	Materials and Methods – Study Design and Setting; Participants; Variables
Participants	6(a)	Give the eligibility criteria, and the sources and methods of selection of participants. Describe methods of follow-up.	Materials and Methods – Participants (criteria 1–10); Supplementary Figure S1. REV1: rationale added for restricting the cohort to elective degenerative indications
	6(b)	For matched studies, give matching criteria and number of exposed and unexposed.	Not applicable (single-cohort design without matching)
Variables	7	Clearly define all outcomes, exposures, predictors, potential confounders, and effect modifiers. Give diagnostic criteria.	Materials and Methods – Variables; Outcomes. REV1: new subsection Definition of "Uncomplicated"
Data sources / measurement	8	For each variable of interest, give sources of data and details of methods of assessment (measurement).	Materials and Methods – Variables; Supplementary Table S8
Bias	9	Describe any efforts to address potential sources of bias.	Materials and Methods – Participants; Variables; Statistical Analysis; Surgical Procedure and Perioperative Protocol; Discussion – Limitations
Study size	10	Explain how the study size was arrived at.	Materials and Methods – Sample Size; Supplementary Table S7 (achieved precision as bootstrap intervals)
Quantitative variables	11	Explain how quantitative variables were handled in the analyses.	Materials and Methods – Statistical Analysis; Outcomes (age dichotomized at 70 years); Sample Size (n < 30 exploratory flag)
Statistical	12(a)	Describe all statistical methods,	Materials and Methods – Statistical Analysis

methods		including those used to control for confounding.	(Approaches A–C)
	12(b)	Describe any methods used to examine subgroups and interactions.	Materials and Methods – Statistical Analysis
	12(c)	Explain how missing data were addressed.	Materials and Methods – Missing Data. REV1: Supplementary Table S9 with Results – Sampling Density and Denominators; Supplementary Table S10 with Results – Missingness Sensitivity Analysis
	12(d)	If applicable, explain how loss to follow-up was addressed.	Not applicable to the biomarker outcome (no follow-up beyond POD 14; per-timepoint denominators reported). Infection ascertainment: Materials and Methods – Participants; Results – Patient Flow and Baseline Characteristics; Discussion – Limitations
	12(e)	Describe any sensitivity analyses.	Materials and Methods – Statistical Analysis; Results – Subgroup Analyses. REV1: Supplementary Table S10 with Results – Missingness Sensitivity Analysis; Supplementary Table S11 with Results – Within-Patient Change from Individual Baseline
Results — Participants	13(a)	Report numbers of individuals at each stage of the study.	Results – Patient Flow and Baseline Characteristics; Supplementary Figure S1
	13(b)	Give reasons for non-participation at each stage.	Results – Patient Flow and Baseline Characteristics; Supplementary Figure S1
	13(c)	Consider use of a flow diagram.	Supplementary Figure S1
Descriptive data	14(a)	Give characteristics of study participants and information on exposures and potential confounders.	Results – Patient Flow and Baseline Characteristics; Table 1
	14(b)	Indicate number of participants with missing data for each variable of interest.	Table 1 and its footnote: baseline ESR available for 213 of 214 procedures; height for 211 and weight and BMI for 210 (summarized available-case); one procedure's smoking-status and five comorbidity-history fields, and one humeral-fixation label, coded to the institutional default; all other variables complete (N = 214). Per-timepoint biomarker denominators in Tables 2 and 3, audited in Supplementary Table S9
	14(c)	Summarise follow-up time.	Materials and Methods – Variables (preoperative window through POD 14); Tables 2 and 3. Infection ascertainment to 90 days except the six most recent procedures — see item 12(d)
Outcome data	15	Report numbers of outcome events or summary measures over time.	Results – CRP Trajectory; ESR Trajectory; Tables 2 and 3
Main results	16(a)	Give unadjusted estimates and, if applicable, confounder-adjusted estimates and their precision (e.g., 95% confidence interval).	Tables 2 and 3; Supplementary Table S7 (bootstrap 95% CI); Supplementary Table S1
	16(b)	Report category boundaries when continuous variables were categorised.	Materials and Methods – Outcomes; Statistical Analysis; Table 1
	16(c)	If relevant, consider translating estimates of relative risk into absolute risk for a meaningful time period.	Not applicable (descriptive reference curves; no relative-risk outcome)
Other analyses	17	Report other analyses done — e.g., analyses of subgroups and interactions, and sensitivity analyses.	Results – Subgroup Analyses; Figure 3; Supplementary Tables S2–S5; Supplementary Table S6 (convergence diagnostics)
Discussion —	18	Summarise key results with reference to	Discussion – Main Findings

Key results		study objectives.	
Limitations	19	Discuss limitations of the study, taking into account sources of potential bias or imprecision.	Discussion – Limitations
Interpretation	20	Give a cautious overall interpretation of results considering objectives, limitations, multiplicity of analyses, results from similar studies, and other relevant evidence.	Discussion – Clinical Implications; Clinical Application and Caution
Generalizability	21	Discuss the generalisability (external validity) of the study results.	Discussion – Limitations; Future Directions
Other — Funding	22	Give the source of funding and the role of the funders for the present study and, if applicable, for the original study on which the present article is based.	Back Matter – Funding

STROBE — an international, collaborative initiative of epidemiologists, methodologists, statisticians, researchers, and journal editors involved in the conduct and dissemination of observational studies. See www.strobe-statement.org.
